# Melatonin delayed senescence by modulating the contents of plant signalling molecules in postharvest okras

**DOI:** 10.3389/fpls.2024.1304913

**Published:** 2024-03-07

**Authors:** Liyu Shi, Yutong Chen, Wanqi Dong, Saisai Li, Wei Chen, Zhenfeng Yang, Shifeng Cao

**Affiliations:** College of Biological and Environmental Sciences, Zhejiang Wanli University, Ningbo, China

**Keywords:** okra, melatonin, IAA, GABA, GA, ABA

## Abstract

Okra has been widely cultivated worldwide. Consumers appreciate its nutritional value and delicious taste. However, okra is very perishable after harvest because of rapid senescence and high susceptibility to mechanical injuries, which limits its storage life and reduces consumer acceptance. This study examined the influence of melatonin treatment on senescence process and endogenous plant signalling molecules in postharvest okras. The results indicated that melatonin treatment delayed senescence by increasing the endogenous melatonin content through upregulation of its biosynthetic genes. In addition, the treatment increased the contents of indole-3-acetic acid (IAA) and gibberellin (GA) due to the positive modulation of their metabolic and signalling genes. Furthermore, treated okras exhibited higher levels of γ-aminobutyric acid (GABA) but lower abscisic acid (ABA) content, contributing to the delayed senescence process compared to control. Overall, the findings suggested that melatonin postponed senescence in okras fruit by positively regulating endogenous signalling molecules such as melatonin, IAA, GABA, GA, and ABA.

## Introduction

Okra (*Abelmoschus esculentus* L.), also known as ladies’ fingers, is a warm-season vegetable that belongs to the mallow family. It is a popular crop in many parts of the world, particularly in Africa, India, and the southern United States (Mishra et al., 2017). The plant is known for its distinctive green pods, which are long, slender, and contain small edible seeds. It is a good source of vitamins and minerals such as calcium and potassium, which is enjoyed by many people around the world (Tavershima Richard and Iveren Blessing, 2022). However, okra is a highly perishable crop, and proper postharvest management is essential to maintain its nutritional value and marketability (Liu et al., 2017).

Melatonin is a signalling molecule that has been shown to regulate quality and storage life of fruit and vegetables after harvest (Feng et al., 2022). Melatonin treatment could increase the antioxidant capacity and slow down senescence in postharvest strawberries (Liu et al., 2018). The treatment also postponed senescence and maintained the firmness and color of postharvest broccoli florets by regulating the expression of genes involved in antioxidant defense and chlorophyll catabolism (Wu et al., 2021; Lou et al., 2023). Furthermore, melatonin has been reported to improve the resistance to biotic and abiotic stresses such as chilling injury, pathogen infection, and mechanical damage in postharvest horticultural products (Feng et al., 2022). The prior investigations we conducted have revealed that melatonin treatment reduced the severity of chilling injury in postharvest peaches (Cao et al., 2016; Cao et al., 2018).

Recent evidences suggest that melatonin plays a critical role in coordinating multiple signalling pathways in plants, enabling them to respond to environmental stimuli and adapt to changing conditions (Arnao and Hernandez-Ruiz, 2018). Melatonin has been found to enhance the effects of auxins on root development by promoting cell division and elongation (Liang et al., 2017). Melatonin regulated shoot branching and leaf senescence via interaction with cytokinins (Wang et al., 2022). Additionally, melatonin has been shown to interact with abscisic acid (ABA), a hormone involved in stress responses, to modify stomatal closure and water use efficiency (Arnao and Hernandez-Ruiz, 2018). The treatment with melatonin promoted grape berry ripening partially through regulating ABA content (Xu et al., 2018). Guo et al. (2023) have reported that melatonin treatment stimulated the generation of endogenous salicylic acid in kiwifruit, triggering the defense response to chilling stress. In addition, melatonin treatment increased chilling tolerance by promoting γ-aminobutyric acid (GABA) biosynthesis in cold-stored peaches (Cao et al., 2016). To our understanding, however, there was a lack of literature on the influence of melatonin, an essential signalling molecule, on other phytohormones with respect to senescence process in postharvest okras. Our previous study has demonstrated that a correlation between the presence of phytohormones, including indole-3-acetic acid (IAA), ABA, and gibberellin (GA), and the senescence process in postharvest okras (Dong et al., 2023). Therefore, the purpose of the current investigation was to evaluate the regulation of melatonin on endogenous plant signalling molecules in relation to the senescence retardation and storage life extension in harvested okras by examining the levels of IAA, GABA, ABA and GA and their metabolizing genes.

## Materials and methods

### Plant materials and treatment

Fresh okra samples were procured from a market located in Ningbo City, China. Samples of uniform size and maturity, devoid of any signs of disease or mechanical damage, were selected. The okras were randomly assigned into two groups of 180 each, and subjected to immersion in either distilled water or 100 µmol L^-1^ of melatonin for a duration of 30 minutes. Melatonin solution was prepared with distilled water. After all the okras were air-dried at room temperature, they were stored for 12 d at 25 ± 1 °C with 80% relative humidity and sampled every 3 d. Each treatment was performed in three replicates (sixty fruit per replicate). At each sampling point, fifteen fruit per replicate were analyzed for senescence index, and then their peels were collected to measure signalling molecule content and gene expression levels.

### Senescence index

The visual evaluation of senescence index on the surface of ten okras from each replicate was conducted. Senescence was rated according to methods described by Dong et al. (2023).

### Endogenous melatonin, abscisic acid, indolacetic acid, gibberellin and γ-aminobutyric acid levels

The levels of melatonin, ABA, IAA, and GA were measured using commercially available kits with instructions provided by Jiangsu Meimian-industrial Co., Ltd, located in Nanjing, China. Enzyme-linked immunosorbent assay (ELISA) was used and the absorbance was recorded at 450 nm. The extraction and determination of GABA followed the methods outlined by Wang et al. (2019).

### Gene expression analysis

Total RNA was extracted and subjected to reverse transcription using the methodology described by Dong et al. (2023). Gene expression was evaluated via SYBR Green I Master Mix (Vazyme, Nanjing, Jiangsu, China) and specific primers (**Supplementary Table 1**) on a StepOnePlus™ real-time PCR instrument (BIO-RAD, Hercules, California, USA). The sequence information for each gene is provided in **Supplementary Table 2**. Due to the stability of the *ACT* gene as a plant reference gene across all samples and conditions (Qu et al., 2019), *AeACT* was chosen as the internal reference gene for calculating gene expression levels using the 2^-ΔCt^ method in okras.

### Statistical analysis

Experimental data were presented as the mean ± standard errors of three replicates. The statistical comparisons between the control and treatment groups were made using Student’s unpaired t-test (* *p* < 0.05, ** *p* < 0.01, and *** *p* < 0.001).

## Results

### Okra appearance senescence index after melatonin treatment

**Figure 1:**
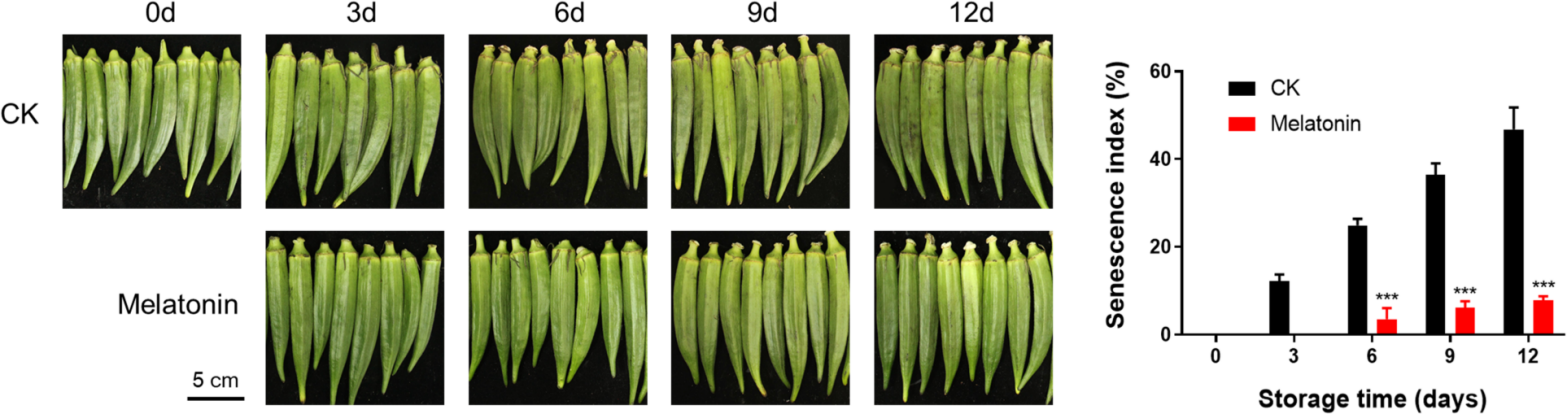
Appearance and senescence index in postharvest okras treated with melatonin during storage. The black bar represents 5 cm. Asterisks indicate significant differences between the control and treatment groups (*** *p* < 0.001).

During storage, the okras without treatment gradually became withered and discolored, as indicated by the rising senescence index. Nonetheless, the application of melatonin treatment substantially postponed the senescence of okras and preserved their quality throughout storage. The senescence index in the treated okras was 81.2% lower than controls (**Figure 1**).

### Endogenous melatonin content and expression of biosynthesis-related genes in okras treated with melatonin

**Figure 2:**
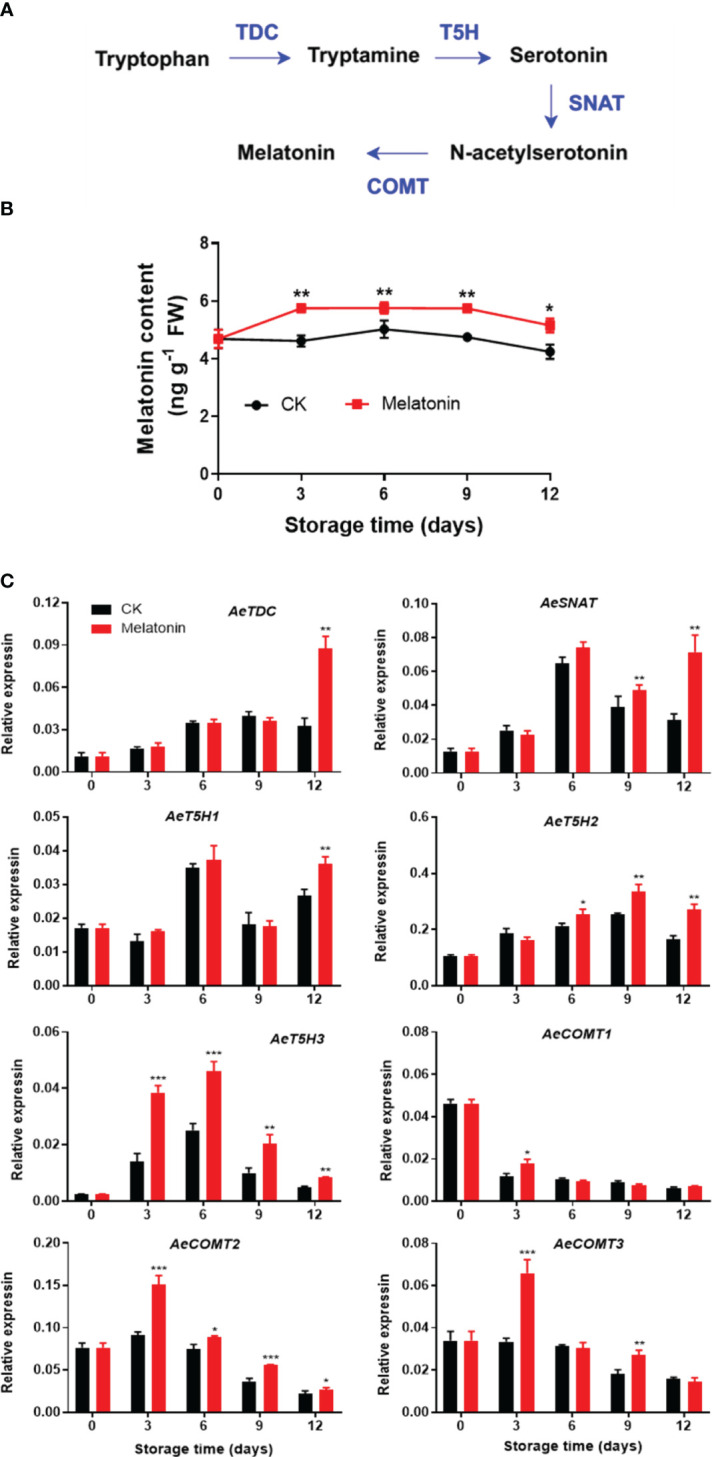
Melatonin metabolism pathway **(A)**, endogenous melatonin content **(B)** and expression of its metabolic genes **(C)** in postharvest okras treated with melatonin during storage. TDC, tryptophan decarboxylase; SNAT, serotonin *N*-acetyltransferase; T5H, tryptamine 5-hydroxylase; COMT, caffeic acid *O*-methyltransferase. Asterisks indicate significant differences between the control and treatment groups (* *p* < 0.05, ** *p* < 0.01, and *** *p* < 0.001).

As illustrated in **Figure 2**, both melatonin-treated and non-treated okras exhibited an initial increase followed by a subsequent decline in endogenous melatonin contents during storage. Nonetheless, melatonin treatment notably elevated the melatonin levels in okras throughout the storage. In parallel, the treatment upregulated the expressions of *AeTDC* and *AeT5H1* at the end of storage. The treated okras displayed higher transcripts of *AeSNAT* and *AeT5H2* after 9 days and 6 days of storage, respectively. Melatonin treatment significantly elevated the expression of *AeT5H3* and *AeCOMT2* throughout the entire storage period. Additionally, the treatment increased the transcript abundance of *AeCOMT3* on days 3 and 9 of storage. Higher expression of *AeCOMT1* was only observed on day 3 in treated okras.

### Endogenous indolacetic acid content and expression of metabolizing genes in okras treated with melatonin

**Figure 3:**
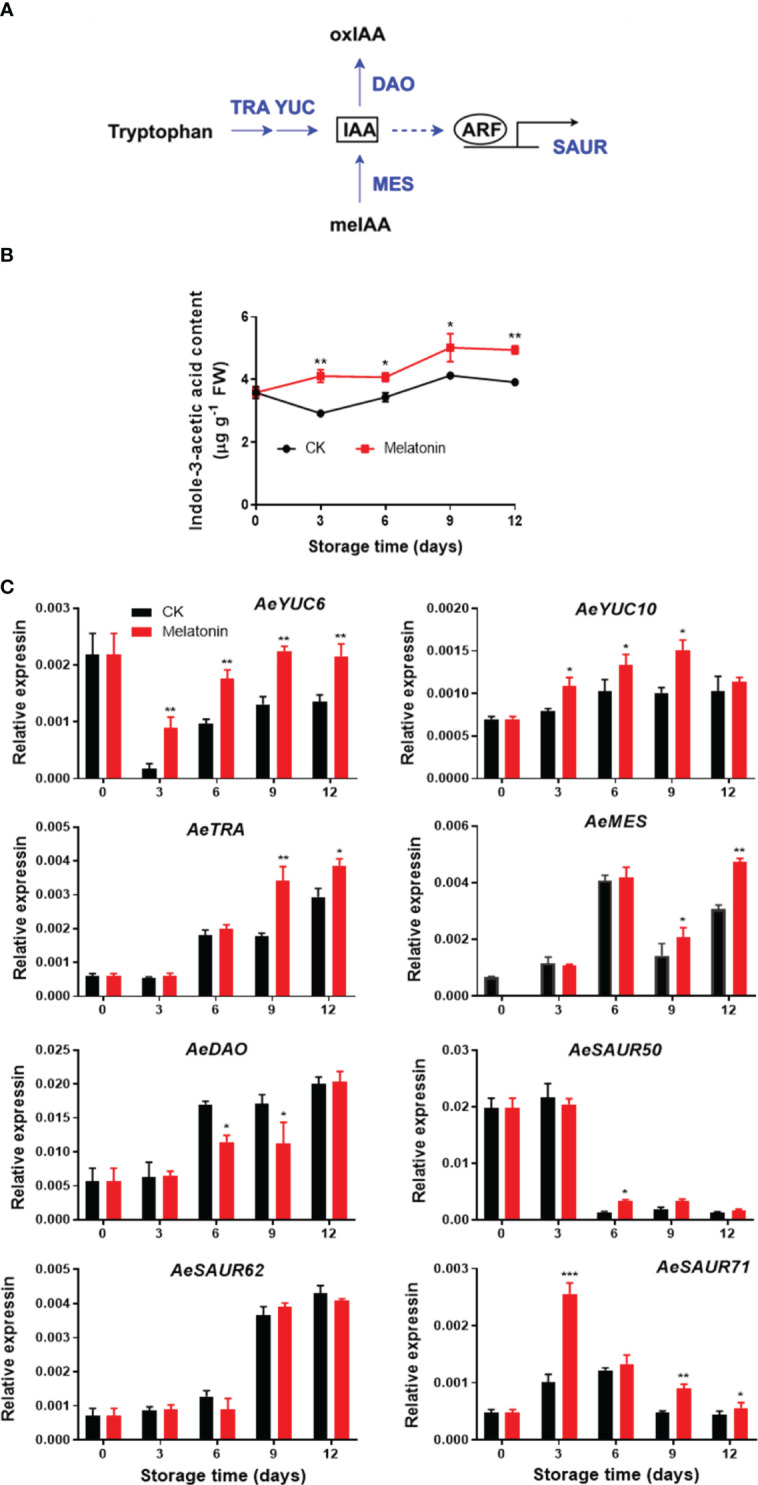
IAA metabolism pathway **(A)**, endogenous IAA content **(B)** and expression of its metabolic genes **(C)** in postharvest okras treated with melatonin during storage. YUC, YUCCA flavin-containing monooxygenases; TRA, tryptophan aminotransferase; MES, methylesterase; DAO, dioxygenase for auxin oxidation; SAUR, small auxin upregulated RNA. Asterisks indicate significant differences between the control and treatment groups (* *p* < 0.05, ** *p* < 0.01, and *** *p* < 0.001).

As illustrated in **Figure 3**, the IAA content in treated and non-treated okras increased gradually during the whole storage with the content in treated okras being higher than that in the controls. The transcript abundance of *AeYUC6* decreased drastically during the first three days of storage followed by an increase towards to the end, which was upregulated by the treatment. Melatonin also increased *AeYUC10* expression after 3 days of storage except day 12. Meanwhile, the okras with treatment also showed higher transcripts of *AeTAR* and *AeMES* after 9 days of storage. On the other hand, the treatment significantly downregulated the expression of *AeDAO*, a gene encoding a protein that degrades IAA, on days 6 and 9. Higher expression of *AeSAUR50* compared to the control was only observed on day 6 in treated okras but no difference in *AeSAUR62* was found between control and the treated okras. Melatonin elevated *AeSAUR71* expression within the storage except day 6.

### GABA content and GABA metabolic gene expression after melatonin treatment

**Figure 4:**
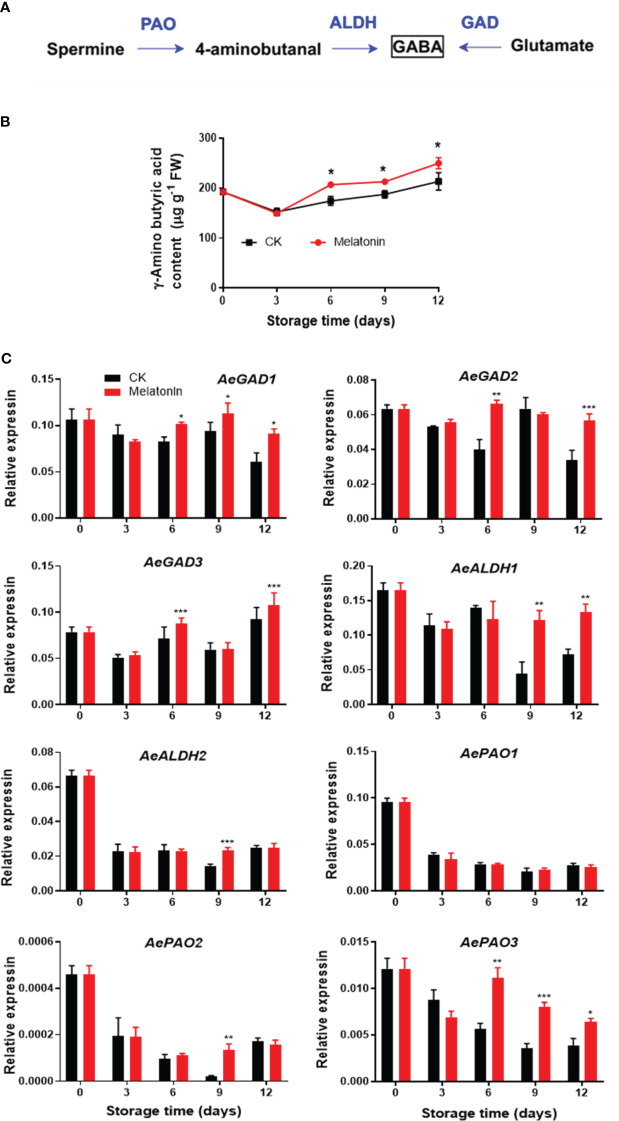
GABA metabolism pathway **(A)**, endogenous GABA content **(B)** and expression of its metabolic genes **(C)** in postharvest okras treated with melatonin during storage. GAD, glutamate decarboxylase; ALDH, aldehyde dehydrogenase; PAO, polyamine oxidase. Asterisks indicate significant differences between the control and treatment groups (* *p* < 0.05, ** *p* < 0.01, and *** *p* < 0.001).

As illustrated in **Figure 4**, GABA content in the control group exhibited a decrease and then an increase during storage. However, melatonin treatment maintained higher level of endogenous GABA content after 6 days. The treatment upregulated the *AeGAD1* expression during the whole storage. The transcription levels of *AeGAD2/3* were increased by the treatment on days 6 and 12. After 9 days of storage, melatonin enhanced *AeALDH1* expression, however, for *AeALDH2* and *AePAO2*, the enhancement was only observed on day 9. There was no difference in *AePAO1* between the two groups but the treatment elevated the transcripts of *AePAO3* after 6 days.

### GA content and GA metabolic gene expression after melatonin treatment

**Figure 5:**
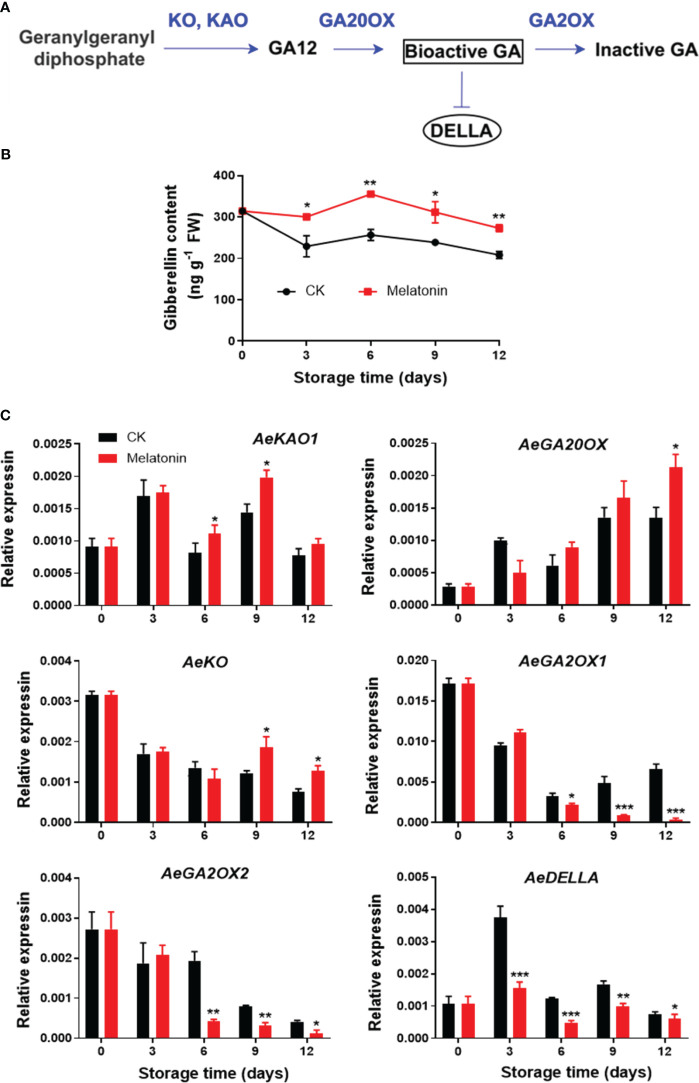
GA metabolism pathway **(A)**, endogenous GA content **(B)** and expression of its metabolic genes **(C)** in postharvest okras treated with melatonin during storage. KAO, ent-kaurenoic acid oxidase; GA20OX, GA 20-oxidase; KO, ent-kaurene oxidase; GA2OX, GA 2-oxidase; DELLA, a key negative regulator of GA signaling. Asterisks indicate significant differences between the control and treatment groups (* *p* < 0.05, ** *p* < 0.01, and *** *p* < 0.001).

As illustrated in **Figure 5**, the GA content in control okras increased gradually firstly and declined during remaining time. The okras treated with melatonin had consistently higher GA levels during the entire storage. The treatment increased *AeKAO* expression on day 6 and 9, and upregulated the transcripts of *AeKO* after 9 days of storage. For genes encoding proteins that degrade GA, melatonin inhibited the expression of *AeGA2OX1/2* after 6 days of storage but elevated *AeGA20OX* expression at the end. During storage, the expression levels of *AeDELLA* was significantly down-regulated with the treatment.

### ABA content and ABA metabolic gene expression after melatonin treatment

**Figure 6:**
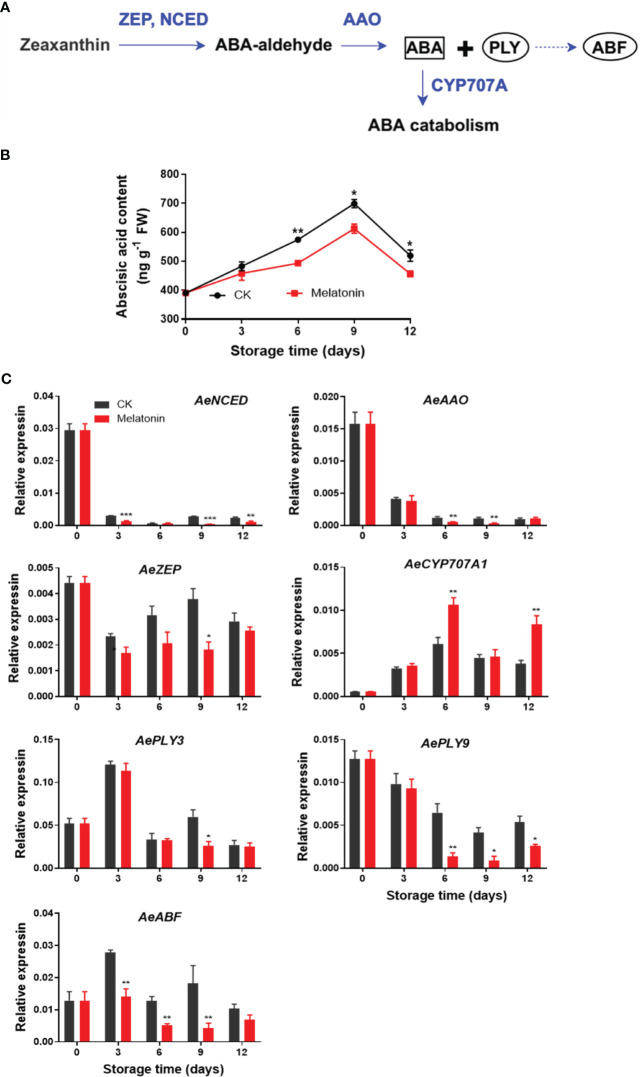
ABA metabolism pathway **(A)**, endogenous ABA content **(B)** and expression of its metabolic genes **(C)** in postharvest okras treated with melatonin during storage. NCED, 9-cis-epoxycarotenoid dioxygenase; AAO, ABA-aldehyde oxidase; ZEP, zeaxanthin oxidase; CYP707A1, CYP707A gene family; PLY, PYR1-like; ABF, Abscisic acid (ABA)-responsive element (ABRE)-binding factors. Asterisks indicate significant differences between the control and treatment groups (* *p* < 0.05, ** *p* < 0.01, and *** *p* < 0.001).

As illustrated in **Figure 6**, ABA content increased gradually in both groups for the first 9 days, then declined at the end of storage. However, okras treated with melatonin had lower ABA content after 6 days. The expression of *AeNCED* and *AeAAO3* in non-treated okras decreased within the storage. Melatonin reduced *AeNCED* expression throughout storage except day 6. The treatment also up-regulated *AeAAO* transcripts on days 6 and 9. Meanwhile, the treated okras also exhibited lower *AeZEP* expression on day 9 but higher *AeCYP707A* expression one of ABA catabolic gene in comparison to the controls. Moreover, melatonin inhibited *AePLY9* expression after 6 days of storage but *AePLY3* on day 9. With the treatment, the transcripts of *AeABF* were down-regulated during the whole storage except day 12.

## Discussion

Melatonin has been shown to mitigate the negative effects of senescence in vegetables by acting as an antioxidant and reducing the levels of ROS (Jayarajan and Sharma, 2021). It also modulated the expression of senescence-related genes, delaying senescence and extending the shelf life in postharvest vegetables (Jayarajan and Sharma, 2021). Various studies have demonstrated the beneficial effects of melatonin on senescence in vegetables, including broccoli, lettuce, and Chinese flowering cabbage (Tan et al., 2021; Wu et al., 2021; Belisle et al., 2023). In the present study, we observed a similar postponement of the senescence process in postharvest okras treated with melatonin. It has been reported that the treatment with exogenous melatonin induced the expression of melatonin biosynthetic genes, thus increasing the levels of endogenous melatonin in postharvest horticultural products (Cao et al., 2016; Hu et al., 2017). Our recent study also found the hydrogen rich water delayed senescence in okras after harvest via upregulating the expression of melatonin synthesis-related genes and endogenous melatonin content (Dong et al., 2023). Therefore, the data presented here clearly showed that suspension of senescence in okras treated with melatonin could be due to the upregulation of melatonin biosynthetic genes and the resultant increase in endogenous melatonin content.

IAA, a plant hormone occurring naturally, participates in numerous aspects of plant growth and development, such as cell division, elongation, and differentiation (Perrot-Rechenmann, 2010). IAA also can regulate senescence by modulating various biochemical and molecular processes (Lim et al., 2010). Senescence in detached leaves of *Arabidopsis thaliana* was associated with a decline in their IAA content due to the decreased transcript abundance of auxin biosynthetic genes (Kim et al., 2011). Exogenous treatment with IAA inhibited the expression of *SAG12*, one of the well-investigated senescence-response genes (Noh and Amasino, 1999), and mutation of the auxin-responsive transcription was beneficial to anti-aging in *Arabidopsis* (Lim et al., 2010). Furthermore, it was reported that melatonin could interact with IAA to regulate plant growth and development (Arnao and Hernandez-Ruiz, 2018). Melatonin treatment increased the levels of IAA in rice and governed root architecture by the upregulation of auxin-related genes (Liang et al., 2017). Acting as a powerful antioxidant, melatonin alleviated leaf senescence by increasing IAA levels via upregulating the expression of genes involved in IAA biosynthesis and signalling in cucumber plants (Jing et al., 2022). The results of our study revealed that melatonin increased the expression of genes involved in IAA biosynthesis including *AeYUC*s, *AeTRA*, and *AeMES*, as well as the auxin response genes (*AeSAUR*s) in okras after harvesting. Conversely, the transcriptional activity of the IAA catabolic gene, *AeDAO*, was suppressed by the treatment. Therefore, the treatment increased the endogenous IAA content due to coordination of its metabolizing genes, which could be considered as one of melatonin reaction mechanism to delay senescence and in postharvest okras.

According to our results, melatonin treatment increased endogenous GABA content which was probably the other reaction mechanisms of melatonin treatment to extend storage time in postharvest okras. In plants, GABA is involved in many physiological processes, including the regulation of growth, development, senescence processes (Khan et al., 2021). Application of GABA postponed the initiation of leaf senescence in *Arabidopsis thaliana* by upregulating the expression of genes involved in antioxidant defense and stress responses (Bouche et al., 2003). Vaezi et al. (2022) found that the improved GABA biosynthesis and accumulation played an important role in decreasing senescence and deterioration rate in strawberries treated with methyl jasmonate. Melatonin has been reported to increase the endogenous GABA levels in yellow-flesh peaches and enhanced the antioxidant defense system, leading to reduced oxidative stress and improved fruit quality (Wu et al., 2023). Our study demonstrated that treatment with melatonin increased the expression of genes involved in GABA biosynthesis, such as *AeGAD*s, *AePAO*s, and *AeALDH*s, resulting in higher levels of GABA content, which was in line with our prior investigation on peaches that exogenous melatonin treatment triggered the accumulation of GABA content through enhanced GABA shunt activity (Cao et al., 2016).

Additionally, we also found that the increased GA content induced by melatonin treatment could be considered as the other reason for the delayed senescence process as compared to control. The positive effect of GA on delaying senescence in plants are well documented. For example, exogenous treatment with GA postponed leaf senescence in Chinese flowering cabbage as experienced by down-regulating the expression of a series of senescence-associated gene (Fan et al., 2018). Treatment with GA_3_ increased the content of endogenous GAs, thereby retarding the chlorophyll degradation and senescence of shoots in *Paris polyphylla* (Li et al., 2010). Furthermore, GA_3_ has been demonstrated to modulate the activity of genes that play a role in chlorophyll breakdown, which is a hallmark of senescence in harvested okras (Xiao et al., 2022). Prior research has demonstrated that melatonin could potentially interact with GA, contributing to plant growth and development beneficially. Melatonin promoted the antioxidant systems and GA biosynthesis to enable seed germination in high salinity conditions in cucumber (Zhang et al., 2014). The interaction between melatonin and GA_3_ has also been linked to the regulation of stress induced senescence in plants in which melatonin pre-treatment modulated GA-mediated pathways to suppress heat-induced senescence in tomatoes (Jahan et al., 2021). In this study, the transcription abundance of *AeKAO*, *AeGA2OX* and *AeKO*, three essential genes responsible for GA synthesis, was induced with melatonin treatment after 6 days of storage. In addition, we also found that melatonin treatment down-regulated the degradative genes *AeGA20X*s and negative regulatory factor DELLAs. However, it is worth noting that the three GA biosynthetic genes were upregulated starting from the 6th day after treatment, while GA began to accumulate on the 3rd day. Therefore, GA upregulation might not be a result of the enhanced biosynthesis during the early stage of storage. However, Zhou et al. (2022) found that over-expression of yam *DoDELLA1* in tobacco resulted in reduced GA content, indicating a feedback and feed-forward mechanism of DELLA on GA levels. Interestingly, in our study, the inhibition of melatonin on the expression of *AeDELLA* started from day 3, which could be the reason why the treated okras displayed higher GA content on the 3rd day. Taken together, our results suggested that the increase in endogenous GA content and signalling could involve in melatonin-mediated the delay of senescence in postharvest okras. However, the detailed feedback regulation of *AeDELLA* on GA content in melatonin-treated okras should be investigated in the near future.

ABA is a hormone that regulates plant responses to environmental stresses, such as drought, salinity, and extreme temperatures (Hong et al., 2013). ABA also contributes to regulate the degradation of chlorophyll and onset of senescence, leading to the yellowing of leaves and the eventual death of the plant. Gao et al. (2016) found ABA promoted chlorophyll breakdown and leaf aging through transcriptional activation of senescence-associated genes and genes involved in chlorophyll breakdown in Arabidopsis. The interaction or cross talk between melatonin and ABA has been reported to play an important role in affecting the responses to biotic or abiotic stresses in plants (Arnao and Hernandez-Ruiz, 2018). The induction of drought tolerance and suppression of leaf senescence in apples treated with exogenous melatonin was related to the regulation of ABA metabolism (Li et al., 2015). Melatonin treatment delayed leaf senescence through inhibiting ABA accumulation and maintaining chlorophyll contents via down-regulating ABA signaling transcription factors in Chinese flowering cabbage (Tan et al., 2019). The decrease in ABA biosynthesis and downregulation of signalling pathways was also contributed to the suppression of heat-induced leaf senescence by exogenous melatonin in perennial ryegrass (Zhang et al., 2017). Based on our experiment results, melatonin treatment was found to decrease the expression of ABA biosynthetic genes, such as *AeNCED*, *AeZEP*, and *AeAAO*, while increasing the transcripts of degradative gene *AeCYP707A*. As a result, the ABA content was reduced, which ultimately led to the inhibition of senescence in okras treated with melatonin. Previous studies have reported PLYs and ABFs were involved in the regulation of ABA-mediated leaf senescence in plants (Zhao et al., 2016; Tan et al., 2019). Similarly, in our present study, *AePLY3/9* and *AeABF* were also downregulated by melatonin. These findings suggested that melatonin had the potential to regulate signals associated with ABA, thereby slowing down the senescence process in okras after harvest.

## Conclusions

**Figure 7:**
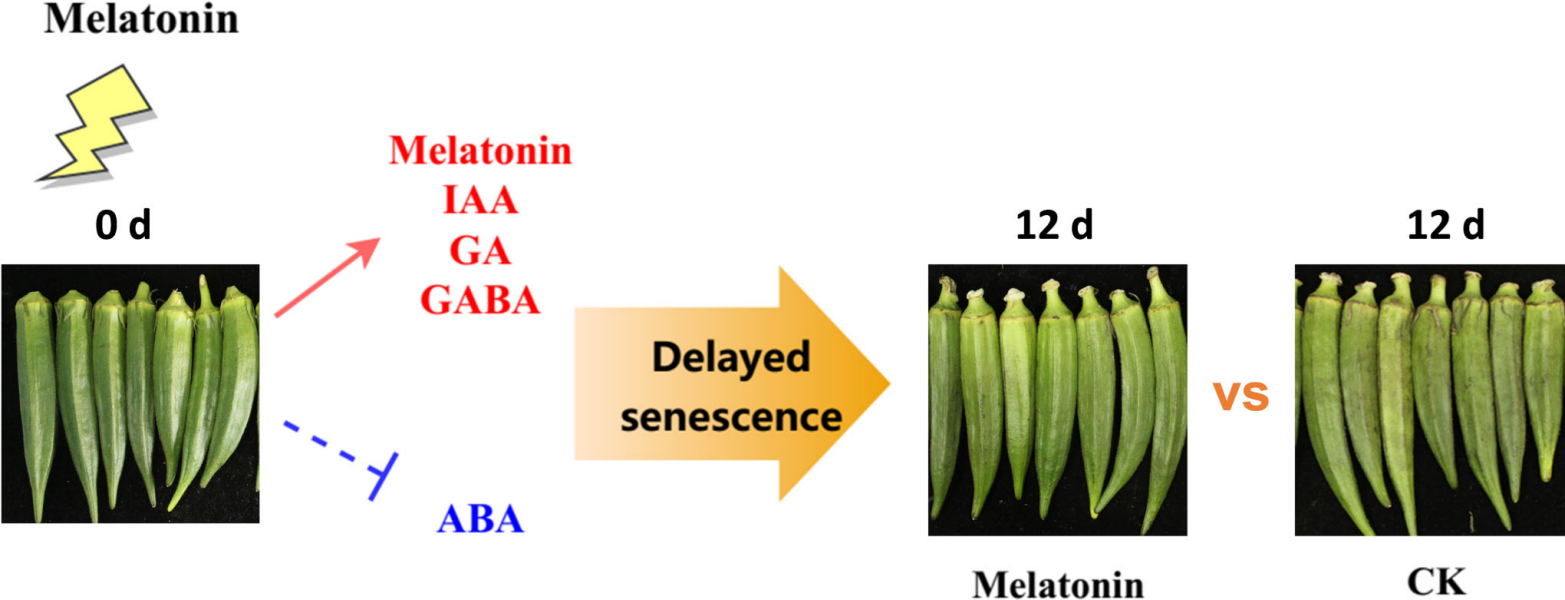
Experimental framework. Melatonin treatment positively regulated endogenous plant signalling molecules including melatonin, IAA, GA, and GABA, and inhibited the accumulation of ABA, thereby delaying senescence in okras after harvest.

In conclusion, our results showed that melatonin treatment delayed senescence in postharvest okras. The coordination of gene expression involved in plant signalling molecules pathways by melatonin led to the increased levels of endogenous melatonin, GABA and GA but decline in ABA content, contributing to the suppression of senescence process in treated okras (**Figure 7**). However, melatonin has demonstrated the ability to regulate diverse physiological processes in plants by interacting with other signaling molecules, such as hydrogen peroxide and nitric oxide. Therefore, additional investigation is necessary to fully underpin the regulation of cross-talk and transduction interactions to reveal the mechanism in the positive effect of melatonin on senescence in horticultural products.

## Data availability statement

The original contributions presented in the study are included in the article/**Supplementary Material**. Further inquiries can be directed to the corresponding author.

## Author contributions

LS: Investigation, Methodology, Writing – original draft. YC: Resources, Visualization, Writing – original draft. WD: Resources, Software, Writing – original draft. SL: Data curation, Validation, Writing – original draft. WC: Supervision, Validation, Writing – original draft. ZY: Funding acquisition, Project administration, Writing – review & editing. SC: Conceptualization, Writing – review & editing.
