# Supplementary material for: Melatonin delayed senescence by modulating the contents of plant signalling molecules in postharvest okras
Source: Front Plant Sci. 2024 Mar 7;15:1304913. doi: 10.3389/fpls.2024.1304913 (PMC10954822; doi:10.3389/fpls.2024.1304913)
Supplement: Supplementary file 1 [file Table_1.docx]

**Supplementary Table 1. Primer sequences used for RT-qPCR analysis**

| **Gene name** | **Forward Primer** | **Reverse Primer** |
| --- | --- | --- |
| *AeTDC* | GGAGAAGCAAGGAAGATT | AATGGTGTTGGAATAGTGA |
| *AeSNAT* | ATAGACTTGAAGCACTTG | CCGTAGACAGAAGACTAA |
| *AeCOMT1* | GCTCTGAGTCTTATGAATC | GCCTTGTTGAATGGAATT |
| *AeCOMT2* | CTACTCCATTGTTACCTG | ACACCATCTTCATTCTTG |
| *AeT5H1* | TGATGCTGGGAATGGAAA | GAGAACTGCCTTGATATGATT |
| *AeT5H2* | CAAGACCTATGATGTTACC | ACCAAGTTATATCGGAGTA |
| *AeT5H3* | GTTGTATGCTCTTGATATGG | AGTTCACTTGGCTTCATC |
| *AeGA20OX* | GGCATAGGCAGAGCACATT | TGGCAAGGAGGATAGTAATTCAAC |
| *AeKAO* | CAGAAGCATTCACTTGGAT | TTGGCGTTGTAGTCATTC |
| *AeGA20X1* | TTGAATCTGAAGCCACAA | ACCAACATCACCATTAGG |
| *AeGA20X2* | CTCAAATCCTTTCTCTCA | GCTATCAGTTCCAGTATT |
| *AeKO* | GGCTTCTACTGTTATTGT | ACTCATTGTAATCACTTGT |
| *AeDELLA* | TGAAGTTGAGTCATTGTTG | AAGTAATAGAGCCACCAC |
| *AeYUC6* | ACTACACCTTCACTTACC | CGACGAATTGTTGCTTAG |
| *AeYUC10* | GCTACTTGGACGATTACG | ATTCCTCGGTCTCTTCAT |
| *AeTAR* | ATATCCGACAACAGTGAA | ACCTCTTGGACATAACAT |
| *AeSAUR71* | TGTTGAGGATTCCGATTA | AAGGCTTAAAGGTTGTTG |
| *AeDAO* | CAAGCCTCTCCTTCACAA | GCAGTAATCTCCATTCAATCC |
| *AeMES* | GATGATTATAACCAGCCTCTC | CAGCACTATGACCTACCA |
| *AeNCED* | TCCATCGTCTTCTTCTATTCC | GATTCCATTGCTGAGATTGAG |
| *AePLY3* | TTGGAAGTGTAAGAGAAG | ACAATGGATGAGTAGTTC |
| *AeZEP* | GCAGACTATTATTGGAGAC | CGAAGGTATCACTATTGAC |
| *AePLY9* | TGTTCCTCTGCTCTTGTC | TTATACTTCTGCGGCTGAT |
| *AeAAO* | AAGATGGAGACGAGAAGA | CCTGGATTATCAGCAACA |
| *AeABF* | CAACAGCATAGATACTTC | GTTCTTAATCATTCTCCTT |
| *AeCYP707A* | ACCATCATCTCTAACATTG | TCATCCTCTCCGAATATC |
| *AeALDH1* | ATGGCAACTCGCAGAATC | AAGCAGAGAAGTAGAGACAGA |
| *AeALDH2* | CCATTGCTGCTAAGATAACA | GTCCCAAACTGCTTCATC |
| *AePAO1* | GGTATTGACATCCGCTTGA | TCCATCTTCCACAGTAATCTTC |
| *AePAO2* | GGTAATCATCATCATAATCA | ATAGCATCTGTCTTGTAA |
| *AeGAD1* | AGTATGGTCTTGTCTATG | AAGGTAGTTGATGTGAAA |
| *AeGAD2* | AACTGGCTATGGCTGAAG | TGTTCTTGCTGGATATTCTCT |
| *AeACT* | TCGGTGAGAAGCACAGGGTG | GGTTGGGATGGGTCAGAAGG |
