# Supplementary material for: Melatonin delayed senescence by modulating the contents of plant signalling molecules in postharvest okras
Source: Front Plant Sci. 2024 Mar 7;15:1304913. doi: 10.3389/fpls.2024.1304913 (PMC10954822; doi:10.3389/fpls.2024.1304913)
Supplement: Supplementary file 2 [file Table_2.docx]

**Supplementary Table 2**

>*AeTDC*

ID: Unigene0007993

CDS:

ATGGTGTTATCTAAGACAGCATCTCAGAGTGATGTCTCTGTTCACTCCACTTTTGCCTCCCGTTATGTCCGGGACTCACTTCCCAGGTTTAAGATGCCAGAAAACTCTATACCAAAGGAAGCCGCGTTTCAGATCATCAACGATGAACTCATGCTGGATGGGAACCCGAGGTTGAACCTTGCCTCGTTCGTTACCACATGGATGGAGCCGGAATGTGATAAGCTTATAATGGATTCCATTAACAAGAACTATGTGGACATGGATGAGTACCCTGTCACCACTGAGCTTCAGAATCGTTGTGTGAACATGATAGCTCACTTGTTTAACGCGCCATTGGGGGATGTTGAGGCTGCCGTTGGGGTCGGAACGGTCGGTTCGTCTGAGGCAATCATGTTGGCTGGCCTTGCTTTCAAGAGGAAATGGCAAAACAAGCGTAAAGCTGAAGGGAAACCTTATGATAAGCCAAACATCGTGACCGGTGCCAATGTTCAGGTCTGCTGGGAGAAATTCGCAAGGTACTTTGAAGTGGAGTTGAAAGAAGTGAAGCTTAGAGAAGGCTACTACGTCATGGACCCTGTCAAAGCAGTGGAAATGGTCGATGAAAACACCATCTGTGTAGCTGCAATCTTGGGTTCAACTCTCAATGGTGAATTTGAAGATGTCAAACTCCTAAACGATCTCTTAACAGAAAAGAACAAACAAACTGGATGGGATACCCCCATTCATGTTGATGCAGCTAGTGGTGGATTTATTGCACCATTTTTGTACCCAGAACTGGAGTGGGACTTTAGGCTTCCCCTTGTGAAGAGTATTAATGTCAGTGGGCATAAATATGGTCTTGTTTATGCTGGTATTGGTTGGGTTATCTGGAGAAGCAAGGAAGATTTGCCTGAAGAACTCATCTTCCATATTAACTATCTTGGAGCTGATCAACCCACTTTCACTCTCAATTTCTCCAAAGGTCACTATTCCAACACCATTTAATATATATGTAGGTGTATATAAACAGCAAAACTGAACTAGTGTTTTACATATCCGCAGGTTCTAGCCAAGTCATTGCTCAGTACTACCAAC

>*AeSNAT*

ID: Unigene0090139

CDS:

ATGGAAGACTCCTCTTCTGTTCAATTGCATCAAAACAAACCCCAGGATGAATGCTCAAACCTGTCTCTTCGGCCATTAGATGTTTCAGACATAGATGCTTTCATGGTCTGGGCTACTGACGAGCAAGTGACACGCTTTTGCACATGGGAGCCTTACGCTAACAAAGAAGATGGCTTGAATTACATCAAGAACATTGTATTGCCTCACCCTTGGTTCCGAGCAATATGCGTCAACGACCAACCGATCGGGGCTATTTCGGTGACATCGAATTCAGGGAACGATAGGTGCAGGGGCGAGCTCGGGTATGTTCTGGCGTCTAAGTACTGGGGCAGAGGGATTGCGACGAGGTGTGTGGGACTGGTGTCTGAGACCATATTTGATGAATGGCCTCATTTGGATAGACTTGAAGCACTTGTTGATGTGGACAATTTGGGGTCTCAAAGGGTATTGGAGAAGGCAGGGTTCCAAAGGGAAGGTGTTTTGAGGAAGTATGTTGTTTTGAAGGGAAGGTCTCGAGATATGGTTATGTTTAGTCTTCTGTCTACGGATCATCGTCGA

>*AeCOMT1*

ID: Unigene0007483

CDS:

ATGAGTTCAACAGGTGAGACTCAGATGACTCCGACCCAAGTCTCAGACGAGGAGGCCAACTTGTTCGCCATGCAACTCACCAGTGCATCGGTCCTGCCCATGGTCCTCAAATCAGCCATAGAACTAGACCTGCTGGAGATCATGGCCAAGGCTGGTCCAGGTGCTTTCCTTTCCCCGAAAGAAGTGGCCTCCCAGCTCCCCACCACCAATCCTGACGCACCGGTTATGCTGGACCGCATCCTGCGCCTTCTTGCCAGCTACTCCATCCTCACCTGCTCCTTGCGCGATCTCCCAGATGGCAAAGTCGAGAGACTCTACGGCCTTGGCCCTGTCTGCAAATTCTTGACCAAGAATGAAGATGGTGTCACACTTTCAGCTCTGAGTCTTATGAATCAAGACAAGGTTCTTATGGAGAGCTGGTACTACTTGAAAGAAGCAGTGCTGAAAGGTGGAATTCCATTCAACAAGGCCTACGGAATGACCGCGTTTGAGTACCATGGCACGGATCCTAGATTCAACAAGGTTTTCAACAGGGGGATGTCTGATCACTCCACCATTACCATGAAGAAGATTCTCGAGACCTATGATGGCTTCGAGGGGCTCAAAACGCTGGTCGATGTCGGTGGTGGTACTGGAGCCACGCTTAACATGATTGTCACCAAGCACCCTTCCATAAAGGGCATTAACTTTGATTTGCCTCATGTCATCGAGGATGCCCCGGCTTATCCTGGTATGACCTTGTCCATG

>*AeCOMT2*

ID: Unigene0037356

CDS: CTTTCCACGATAGAAGTGGCTGCACAGCTCCCCACCACAAACCCTGATGCTCCGGTTATGCTGGACCGCATCCTGCGCCTACTCGCCAGCTACTCCATTGTTACCTGCTCCTTGCGTGATCTCCCCGATGGGAAAGTCGAGAGACTCTACGGCCTTGGCCCTGTCTGCAAATTCTTGACCAAGAATGAAGATGGTGTCACTCTTTCCGCACTGAGTCTCATGAATCAAGACAAGGTCCTTATGGAGAGCTGGTACTACTTGAAGGATGCAGTGCTGGAAGGTGGAATTCCGTTCAACAAGGCCTATGGAATGACTGCATTCGAGTACCATGGCACGGATCCTAGATTCAACAAGGTTTTCAACAGGGGGATGTCTGATCACTCCACCATTACCATGAAGAAGATTCTC

>*AeT5H1*

ID: Unigene0039306

CDS: ATGGGTTTGTTTAATTCACTCCTTTGGCTTTCATTTCTCCTCCTTCCCCTGTCGCTACTCTTCAAGAATATGACCGATCGAGTTAACAAAAAACTCAAGAATCTCCCACCGAGCCCACCCAAGCTACCGATCATAGGCAACTTGCACCAGCTCGGTGCACTGCCACACAATTCTCTCCGACAACTCGCTCGAGAATATGGTCCAGCCATGCTCCTTCATCTAGGTCGTATACCGGTCCTCGTCGTTTCATCGGCCGAAGCGGCCAAGGAATTGTTAAAGGACAATGATCTTGCTTGTTGTAGTCGGCCTAAGTTAGCTAGTGTCGGGAGGCTTTCGTATAATTATTTAGACGTAGCGTTCTCGCCATACAATGAGTACTGGAGAGAGTTGCGGAAACTATGTGTTCTTGAGGTTTTTAGTGTCAAAAGAGTGAAGTCTTTCCGGTACTTAAGAGCAGATGAGGTCGGGTCGTTAATGGATTCGATCGCTCGGTCGGCATCGGCGTTCCCGAATGATCCGGTGAATTTGACCGAGAAGGTGTTTGCTTTGACTGGAAGCATAATATTCAGGAAAGCTTTCGGTAAGAGCTTCCATGGAAGCGAGTTTGATCGTGGTAAGTTCTACGAATTGGTTCACGATGCCGAGACGGTGGCCGGAGCTTTCTCGTACGCCGAATGTTTTTCGGGATTTGGCTGGATTATGGACTGGTTAAGTGGTCATAATGAGAGAGTGGAGAGAGTTTTCCGTGAGCTGGATGCGTTGTTTCAACAGGCGATCGATGAACATCTGAAACCTGGGAGGACAAACCATGGGGAAGACATTATTGACGTGATGCTGGGAATGGAAAAGGAGCAAACCGAAGAGCATGGTCGGGCTTGGATAACTAAGAATCATATCAAGGCAGTTCTCTTGAACATGTTCTTGGGTGGCATAGACACCAGCGCGCTTACCGTAGAATGGGCAATGGCGGAGCTTATGAGAAAGCCAACACTGATGAAGAAAGCACAAGCCGAGGTTCGAGGGATCGTGGCCGAAAAAGGAAGAGTACCGGAAGCCGATCTCGACCAACTTCAATACCTAAAGATGATTATCAAAGAAACTCTAAGATTACATCCTCCGGCGCCGATGTTGATCGCCAGAGAATCGATTTCACACTTCAAAATCAAGGGCTACAACATTTATCCCAAAACACTTATCCAAGTCAATGCTTGGGCCATAGGAAGGGACCCAAAGAACTGGGAAAACCCAGAAGAATTCTCCCCGGAAAGGTTCATCGGCAACTCCGGTGATATCAAAGGACAGAATTTTGAATATTTGCCATTCGGAGGTGGCAGACGAGGGTGTCCGGGGATATACATGGGAACCGTCACGTCAGAGTTTTTACTCGCGAATCTCTTGTACTGTTTCGACTGGGCTCTGCCGGTTGGGATGGAAGCGGGTGATATTAACATGGAAGAGAAGGCCGGTCACTGCCTTACCCTGTCGAGAAAAACACCTCTTCTCCTTGTGCCGATCAAATACCATAATGGTTAGCCATCT

>*AeT5H2*

ID: Unigene0112667

CDS: GAAACACCATCTTGGGCTCCTTACTTCACAGCTTGGCTAGCCACTCTAGCACTCATCCTCGTCTCCCTCCGTCTCCGCCGCCGTGAAAAGCTCTACTTGCCGCCTGGACCGAAAGCATGGCCCATAATAGGCAACCTTAATCTAATTGGTTCGCTTCCTCATCGGTCCATCCATGTTCTCTCCCAAAAGTATGGTCCTATTATGCAGCTCAAATTCGGGTCATTTCCTGTTGTGGTGGCTACGTCCGTTGATATGGCCAAAGCGTTCCTCAAGACCTATGATGTTACCTTTGTCGATCGGCCTAAGATTGCGGCCGGGGAATTCACTACTTACAATTACTCCGATATAACTTGGTCACCATACGGACCGTACTGGCGTCAAGCACGAAAAATGTGCATGACCGAACTTTTTAGTGCGAAACGTTTGGAGTCGTATGAGTATATCCGGAGAGAAGAAATGAAATTGTTGTTGAAAGGGTTATATGAATCATCCGGTAAGGCGATTGTTTTGAAAGACAATCTTTCTGATTTGAGTCTTAACGTGATAAGCAGGATGGTGTTGGGAAAAAAGTACACGGATGGAACCGGTGAAAATGAAATCGTGACCCCGAAAGAGTTCAAGGAGATGCTGGACGAGTTGTTCCTGCTGAACGGGGTGCTGGACATCGGGGACTCGATTCCCTGGTTAAGATTCCTTGATTTGCAAGGCAATATTAAGAGAATGAAGGCGTTGAGCAAGAAATTCGACAGGTTCTTGGAACATGTTTTGGATGAACATGATGCTAGGAGAAGAGAGATCAAAGATTATGTTGCCAAGGATATGGTGGATGTGCTGTTGCAGCTTGCAGAGGATCCCAATCTTGATGTCAAGCTGGAAAGGCATGGAGTCAAGGCATTTAGTCAGGTAATATGGACCAATGACTTTATAGTTTATACCTACAGATTACAGAACGTAAGCTGATGTTTGATGATGTTCCAGGACCTGATAGCCGGAGGAACCGAGAGTTCAGCCGTGACCGTGGAATGGGCCATTTCCGAGATGTTGAAAAAGCCGGAGATCTTTTCCAAGGCGACGGAAGAACTAGACAGGGTCATCGGCAGGGAACGATGGGTCGAAGAAAAAGACATTGTCAACCTACCCTACATCGAATCAATTGCCAAAGAGACCATGCGTTTACACCCTGTAGCACCTATGCTTGTGCCTCGCATGACCCGTGAAGACTGTCGAGTAGCAGGTTACGACATTCGCAAGGGCACTAGAGCCCTTGTGAATGTATGGACTATTGGGAGAGACCCTACAATTTGGGACAACCCCAATGAATTTTTCCCAGACAGATTCATTGGTATGTCCATCGACGTAAAAGGTCACGATTTTGAGCTGTTGCCGTTCGGAGCCGGAACAAGGATGTGCCCTGGATATCCCCTCGGGATTAAGGTCATTCAAGCCAGTTTGGCTAATCTTTTGCATGGATTTACATGGAAACTGCCTAGTCACTTGAAGACAGAAGATCTGAATATGGAGGAAATCTTTGGTCTGTCCACCCCAAAAAAGTACCCACTGGAGACTGTGGCTGAGCCTAGGCTCCCTCTTCATCTGTAC

>*AeT5H3*

ID: Unigene0004774

CDS: ATGTCTCTAAAACAAGCCATGGATTTGTCTTTCCTACAAACCCTGGAACCATTACCCATGGCACTTCTTTTCCTTACACCCTTCCTTCTGTTTGGCCTCATCTCTCGTCTCCGTAGAGGACCATTCCCTCCGGGACCCAAAGGGCTCCCCATCATCGGCAACATGATGATGATGGACCAGTTAACTCACCGTGGACTTGCCAAACTCGCTCACAAATACGGCGGCATATTTCATCTTAAGATGGGGTTCTTACACATGGTGGCCATCTCCAATCCCGAAATGGCTCGCCAAGTCCTTCAGGTTCAAGACAACATATTCTCCAACAGGCCGGCCACCATCGCCATCAGTTATCTAACCTACGATAGGGCTGATATGGCTTTCGCTCATTACGGTCCTTTCTGGAGACAGATGCGAAAGCTTAGCGTGATGAAGCTTTTCAGTAGGAAAAGAGCCGAGTCGTGGGAATCGATTCGAGATGAAGTCGACTCACTCGTGAAAGCTGTTGCGTCCAATACTGGGAGAGCTATAAACATGGGTGAGTTGATCTTTAATCTTACCAAGAACATCATTTATAGGGCGGCTTTTGGTTCCATTTCGGAAGAAGGGCAAGACGAGTTTATCAGGATCCTGCAAGAGTTTTCTAAGCTTTTTGGTGCTTTCAATATTGCGGATTTTATTCCTTGGTTCGGTTGGGCTGATCCTCAAGGACTCAACACCAGGCTTGAGAAAGCTCGTGGTGCGTTAGACAAGTTCATCGACGTCATCATCGACGATCACATTGAGAAGAAGAATAAGAACAACGGCCGCTCCGATGACGGTGACACCGACATGGTTGATGATTTACTCACTTTCTACAGTGAAGAAGCAAAAGTAAACGAATCAGAGGATCTACAAAATTCTATCAAATTAACTAGAGACAACATCAAAGCTATTATCATGGATGTGATGTTCGGTGGGACCGAGACGGTGGCGTCGGCGATTGAGTGGGCCTTGGCGGAGCTAATGAGAAGCCCAGAAGATATGAAGAGAGTCCAACAGGAGCTGGCGGAGGTGGTGGGGCTTGACCGCCGTGTGGAAGAATCTGACATGGACAAACTTACCTACCTAAAGTGCTGCCTCAAAGAAACCCTCAGGCTCCACCCACCGATCCCCCTCTTGCTCCACGAGACCGCTGAGGATGCCGTGGTAGCCGGCTATCAAATCCCTGCCAAGTCTCGCGTGATGATCAACGCTTGGGCTATCGGGAGAGATAAGAATTCATGGGAAGAGCCTGATAGTTTCAAGCCATCCAGGTTCTTGAGGGAAGGCGTCCCAGACTTCAAAGGAAGCAACTTCGAGTTCATACCGTTCGGTTCAGGTCGGAGGTCATGTCCAGGAATGCAACTCGGGTTGTATGCTCTTGATATGGCAGTGGCTCATTTGCTTCACTGTTTCACGTGGGAGTTGCCTGATGGGATGAAGCCAAGTGAACTCGACATGAGCGACATTTTTGGACTCACAGCCCCTCGGGCGACTCGACTGTATGCAGTTCCCAAGAAACGCCTGGTCTGCCCACTCTTT

>*AeGA20OX*

ID: Unigene0021554

CDS:

GGCCTCAAAATATACCAATCTCTTCATGCAATGGCGATTGACTGCGCCTCCATGCCACACCATCCGAAAGATGAGCAAAAGCAATTGGTCTTTGATGCCTCTCTGCTCAAGCACCAATCCCACATACCGGAACAATTTATATGGCCCGACGACGAACAGCCTTGCTCTAATCCACCCGAACTCCATGTACCTCTCATCGACCTAGGAGGCTTCCTCTCCGGTGACTCTGTTGCTGCGGCAGAAGCTTTACGGCTTGTCGGGGAGGCTTGTCGGCAGCACGGTTTCTTTCTTGTGGTTAATCATGGAGTGGACGCCACGCTCCTGGCTGATGCTCATAGGTACATGGATGATTTCTTTGAATTGCCACTAGGTGAAAAGCAAAGGGCTCAGAGGAAAGTTGGTGAGCTCTGTGGATACGCCAGTAGCTTCACTGGCAGATTCTCCTCCAAGCTGCCTTGGAAGGAAACACTTTCGTTTCGATACTCGGCGCAGAGAGACGCATCGATGATGGTCGAAGACTACCTTGTTAATAAAATGGGAGATGAATTCAGACAATTCGGGAGGGTTTACCAGAACTACTGCGAGGCCATGAGCAAGCTTTCTCTAGGGATCATGGAGCTTTTAGCCGTCAGTCTAGGCATAGGCAGAGCACATTTCAGGGAATTTTTTGAAGAAAACGATTCAATCATGAGGTTGAATTACTATCCTCCTTGCCAGAAACCAGACCTCACATTAGGCACAGGGCCTCATTGTGACCCGACATCATTAACCATCCTTCACCAAGACCGAGTTGGTGGTCTTCAAGTCTTTGTAGACAACGAATGGCGTTCGATTACTCCGAACTTCGACGCATTCGTGGTTAACATCGGCGACACCTTCATG

>*AeKAO*

ID: Unigene0018466

CDS:

ATGGAAATCGTGGGTTCGATGTGGTTGGTGGTGGCCTTGGCAACACTGGGTGGCTTTGCGTTTTTGAAATGGGTGCTGAAAAGCGTGAATCCTTGGGTGTATGAATCCCGGCTGGGCGATTTGAAGTACTCCCTGCCGCCGGGAGACATGGGTTGGCCTTTCATCGGAAACATGTGGTCGTTCCTCCGAGCTTTCAGGTCCAAGGACCCTGATTCCTTCATGGATTCCTTCGTTTCCAGATTTGGACGCACTGGAATCTACAAGGCCTTCATGTTCGGGAAGCCGAGTGTGATCGTAACATTGCCCGAAACATGTAGAAGAGTGTTGAATGATGATGATGCATTTAAGCCTGGGTGGCCTACTTCCACTGCGGAGCTCATCGGAAAGAAATCCTTTGTCAGTATCCCTTTTGAAGAACACAAGCGTCTCCGACGATTAACTGCAGCTCCCGTCAATGGCCATGAAGCACTCAACCTTTACATCCCCTACATCGAGAAGATTGTGGTATCTACCCTGGACAAATGGTCCAAGAAGGGGAAAATCGAGTTCTTAACCGAACTCCGAAGGCTCACTTTTCGGATAATAATGTATATTTTCCTAAGTTCGGAAAATGAAGAGGTGATGGAAGCTTTGGAGAGGGAGTACACTACTCTCAACTATGGAGTTAGAGCCATGGCAATCAATATCCCAGGATTTGCTTACCATAAAGCCCTCAAGGCTCGGAAAAAACTCGTCGCAGCTTTTCAATCCGTAGTGACCGAGCGGAGAATGCAGAGGAAGACAAATACACACTCCACAAAGAAGAAAGACATGTTGGATGCCCTGATAGATGTTAAAGACGAGAAAGGTGCAACACTGGATGATGAGGAAATCATTGATATCATGTTGATGTACCTGAATGCAGGCCATGAATCTTCCGGCCACACCACAATGTGGGCGGCTGTTTACCTGGAACAACACCCTGAATTTATGAAAATAGCAAAGGAAGAGCAAGAGAGGATTTTGAAGAAAAGGCCACCAACCCAGAAAGGTTTGAATCTCAAAGAAATTCGAGAAATGGAATACCTTTCTAAGGTGATTGATGAAACGCTTCGGTTGGTAACATTCTCCCTGACTGTTTTCCGTGAAGCAAAAACAGACGTCAACATAAGTGGTTATACTATCCCAAAGGGATGGAAAGCACTAGTTTGGTTCAGAAGCATTCACTTGGATCCGAAGATTTATCCGAACCCAAGAGAATTTATTCCTGATCGATGGAATGACTACAACGCCAAAGCAGGAACTTTCCTTCCCTTTGGAGCAGGAAGCAGACTGTGCCCTGGAAATGATCTTGCCAAGCTTGAAATTTCTATTTTCCTTCACCATTTCCTCCTCAACTATCAGCTGGAACGGCTTAATCCCGGGAGTAAGATTAATTATCTACCTCATACAAGGCCTCAGGATAATTGCTTGGCAATAATCAGGAAGAAC

>*AeGA20X1*

ID: Unigene0028704

CDS:

ATGGTGATATTATCAAAACCAGCAATTGAACAGTTCTCTTGTATCAGAAACAAGCAGCCGGCTGCAGCATTGTTCCCTCCGGTTATCCCTGTTGTTGACCTCTCGAAACCGGATTCGAAACACCAGATAATCAAAGCCTGTGAGGAATTTGGATTCTTCAAGGTGATCAACCATGGGGTTCCCATGGAATTCATTTCCAGGCTTGAATCTGAAGCCACAAAGTTCTTTTCTTTACCTTTGTGCGAGAAGGAGAAAACAGGGCAGCCTAAACCTTATGGATATGGGAATAAAAGGATCGGTCCTAATGGTGATGTTGGTTGGGTGGAATATCTACTCCTCATGACCAACCAAGACCCAAATATCTCTACTGAAAACCCAGAAATTTTCAGAGTTGCTTTGAATAATTATATGGCGGCGGTGAAGGAAATGGCGTGTGAGATACTGGAAATGGTGGCGGATGGGCTTAAGATTCAACCGAGGAATGCTTTGAGTAAGCTGATGATGGATGAACAGAGCGACTCTGTTTTCCGGCTAAACCATTACCCTCCCTGCCCAGATGTTCTTCAATCTCTGAACAGCAATGTGATTGGATTCGGCGAACACACTGACCCACAAATCATCTCGGTTCTACGATCAAACAACACTTCCGGCCTTCAAATCTCACTTAAAGACGGATCCTGGATTTCAGTCCCACCCGACCATCACTCATTTTTCATCAACGTCGGTGATTCCTTACAGGTA

>*AeGA20X2*

ID: Unigene0048566

CDS:

ATGGTTGTTCAGTCACAGACATTAACCCATTGTTCTTCAATCAAAACATGCAACAAAGCCGCCGCCGTTTTCACTTCAATCCCAGTGATAAACCTCGGAGACCCCGAGTCGAAGTCTTTGATGGTAAAAGCCTGTGAAGACTATGGATTCTTCAAGGTGGTGAATCACCGTGTTCCAATGGAGTTCATTAACAGACTGGAGCGCGAAGCTATCGGGTTCTTCAATCTGCCTCAGTCTGAAAAAGACAGAGCTGGTCCGCCCGATCCTTTTGGTTATGGCAGCAAGAGGATTGGAACCAATGGTGATGTTGGTTGGATTGAATATCTCCTCCTCAATACCAACCCTCAAATCCTTTCTCTCAAAACCCTCACCGTTTTCCGACAAAACCCAGAAATTTTCCGCTCCGCTGTAAACGACTACATCCAAGCAGTGAAAACAATGGCATATGAAATACTGGAACTGATAGCCGATGGTCTGAAAATCGAGCCGAGGGATGCTCTGAGCAAGCTACTAAGAGACGAGAACAGTGACTCGTGTTTCAGGCTGAATCATTACCCGCCTAGTCCTGAGTCGGAGTCACTGAGTCTGAGTGGAATGACTTTGGTCGGGTTTGGGGAACACACCGACCCGCAGATAATATCTGTGTTGAGATCCAACAACACCTCTGGTTTGCAGATTTGTCTGAAAGATGGTACCTGGGTTTCAGTCCCATCTGATGAAACGTCATTCTTTATCAATGTTGGTGATGCCTTGCAGGTACAGTAGAATCTTTTAAATCTCAATATTTTAAAATGGACCGTCTAATGGTAAGAAACTTGGATCATTCTTAAATTTGAGTCTTGTGAATAAAAAATACAATATAAAACATGTGTCCGACTTTTCTTATACATGTTGCTTACCCTCGTTTGATATGTTCTATTTATGAGTCAGGTACTGACAAATGGTAGGTTCCAAAGTGTTAAGCACAGGGTCTCGGCCAACACCCAGAACTCAAGGTTTTCAATGATCTATTTTGGAGGTCCCCCTCTAAGTCAAAAGATTGTGCCTTTCACTTCGCTTATGGGAAAACAAGAAGAGAGTCTGTACAAGGAGTTCACATGGTCTGAATACAAGACCTCGGCGTACAAGTCAAGGTTGGCTGACTACAGGCTGGGTTTGTTCGAGAAAACCCAGAGTACCAAC

>*AeKO*

ID: Unigene0008456

CDS:

TATGGACCTGTTTATTCCATCAGAACCGGGGCTTCTACTGTTATTGTAATCAATTCTCCCGACACGGCCAAAGAGGCCATGGTGACAAGGTTTTCATCCATTTCAACCCGAAAGCTATCGAATGCACTCAAGATTCTAACTTTTGATAAATGTATGGTTGCAACAAGTGATTACAATGAGTTTCATAAAATGGCCAAACGGTGTTTGCTGACAAATACTTTAGGTTCAAGTGCACAGAGACGACACCGACACCACAGAGATGCTATGATTGAAAATATAATAAGCCGATTTCATGCTTTGCTGAGAGATGATCCTCTCCAATCTGTCAACTTCAGGGAGGTATTCGAGTCTGAACTTTTCGGATTGGCAATGAAGCAAGCATTGGGAGAAGACGTGCAATCGATTTATGTGGATGAACTCGGTACAACTTTCTCAAGAAAACAGATACATAGAGTTCTAGTCCTTGATATGATGGAGGGTGCAATAGATGTAGATTGGCGAGATTTCTTCCCATACTTGAAATGGATTCCTAACGAAAGGTTTGAAACCAATATCCAGCAAAAGCATTTCAGGAGGATGGCATTGATGAATGCCCTCATCAAGGAGCAGATGAAACGAATCGAATCAGGGGAGGAAGTGAATTGTTATCTTGATTACTTGTTAGTGGAAGCAAAGACACTTACAAAGGAGCAACTAGCAATGTTACTCTGGGAAACAATCATTGAAACATCAGATACTACTCTGGTCGCAACAGAGTGGGCAATGTATCAGCTTGCTAAAGATCCAACCCGACAGGATCGTCTCTACCATGAAATAGAAAAGGTTTGTGGTTCTAACAAGGTTAAGGAAGAAAACTTTTCCCAAATCCGATACTTGGATTCAGTTTTCCATGAGACCCTTAGGAAGCACAGTCCGGCTCCACTAGTCCCTTTAAGATATGTTCATGAAGATACTCAAATAGGAGGGTATTCCATACCAGCAGGAAGTGAGATTGCTCTTAATATCTATGGCTGTAACATGGACAAGAATCATTGGGACAACCCTGAAGAGTGGATCCCCGAGAGGTTTATCGATGAAAAATACGACCCTTTGGATTTACACAAGACGATGGCCTTTGGAGCTGGAAAGAGGGCGTGTGCAGGCTCTCTTGAGGCAATGTTGATTGCCTGTGCAGCGATTGGTAGGCTGGTTCAGGAATTTGAGTGGAGCCTGAAAGATGGTGAAGAAGAGAAAGTTGATACGGTTGGGTTGACCTCTCAGAAGCTTCATCCACTGTTTGCCATCCTAAAATCACGAAAT

>*AeDELLA*

ID: Unigene0026495

CDS:

ATGGGGTTTTGGTGGCATAATTTCTATCAGAAATCTAGGAAATTCCTCTCTTCTTCCACCATGAAACAACCCTTTCACTTGCTCGCCGTTCTCCTTCTCAGCCTTTGGCTTCCCCTAGCTTTCCTCCTCGTCGCCAGGCTCTCCTACGTCAACTACGTCTTAAACGCCACCGTGATGGACCATTCGACACCGGAGACGCCGTCTTTCCTATGGTCTTTCTACTTCAATGCCAATCCAGCTCTTATCTATTTTCTCGTCACATCCGCCAGCGTCGTCGCTCTTGCACATGGCTTGACCGGGAAATTCGGGTTGGTGGTGACTGAGTTGTCAACGGATACCATTCATCGGCGGCCCCGTTTGTACATTGCTTGGATCGTTTTATGCATCGTGCAAGTTTCCGTCGGGTTGGGGATCGAAGGAAGCGTGGCGGTAGGCGTTGACGGAGCCGGTTTCGGCGTTAAAAGAAGTTTTCTCAGTAGAATGATATTCTTCTTGGGCTTACATGAGACCATGCTTGTTTGGTTCAGAACAGTGGTGAAACCGGTGGCGGATGACTCAATCTTCGGCAGCGTTAGTGAAGAAAGGTGGATTCATAGGACCGCCATCACGTTGGCAGCCGGTACCCTCTGGTGGTGGAAGCTAAGGGATGAAGTTGAGTCATTGTTGGTGGTTGCTGAGGCTAAGAAAGAACTGTCCCTGGACATTGAAATGGCTGATTTTCTCGGGTGGTGGCTCTATTACTTGACCGTCACAATCGGCATGGTTAGGGTCGTGAAAGCTCTCCTTTGGCTTGGCTTCATCTTGCTTTGTGGAAGGAGAAGAAATGACAACATGGATGAAACAATTAGTGAGGAAGATGATCAAGACAAGGTT

>*AeYUC6*

ID: Unigene0009796

CDS:

ATGGACTGCTTGAGAGAAATACTAGGAAAACAAGCTCATGATCCTGTGTTTATTGAGAAGATGAGTAAGTTGTCCTCGTCGCGGCGTTGCGTATATGTTCCGGGGCCACTTATCGTCGGCGCAGGGCCTTCGGGTCTGGCGGCAGCCGCTTGTTTGAAAGAGAAAGGTGTCCCGAGTGTGATCCTAGAGAGATCTAACTGCATAGCATCTTTGTGGCAGCTCAAGACCTATGATCGACTACACCTTCACTTACCGAAGCAGTTCTGCGAGTTGCCACTCATGGGGTTCCCTACTGATTTCCTTACTTACCCTACTAAGCAACAATTCGTCGACTACTTGGAATCGTATGCTCGGAGGTTCGACATTAGGCCGCGGTTCGATGAGGCGGTCTCGTGTGCCGAATACGACTCGACGGTCGGTTTTTGGCGCGTGAGAAGCGTGGGGTCGAAGGGGATTAATGAGACGGAATACGTGTGCCGGTGGCTGGTGGTGGCGACGGGGGAGAATGCGGAGGCGGTGGTGCCGGAGATCGAAGGAATGGGGGAGTTTGGTGGGGACATAAGGCATACGAGTTTATATAAAAGTGGTGAAGAATTTAGAGGGAAAAGGGTTTTAGTGGTGGGGTGTGGGAATTCAGGCATGGAGGTGTGTTTGGATCTTTGCAATCATAATGCTAAGCCTTCGCTTGTGGTCCGAGACACTGTAAGAAAACATTTTTAACTATGGCTAGTGGCAACTCTCTTTTTTATTACAGAACATCAAATTGTTTCCTAACACTCCTCGATATATCCCTGATCGAAGGTGCACGTCCTCCCGCGAGAGATGCTAGGGACATCGACTTTCGGGTTATCGATGTGGTTGCTCAAGTGGCTGCCCATACAGCTCGTCGACTGGTTCCTGCTTATCGTATCATGGTTGATGCTTGGCGACACGACACGGTTCGGGTTGGAGCGGCCACGATTAGGTCCGCTCCAACTTAAGAATGTGTCCGGAAAGACTCCGGTTTTAGACGTTGGCACGCTGGCCAAAATCAAAAGCGGAGACATTAAGGTATGTCCAAGCATCAAGCAACTAAAACCACATGCTGTGGAGTTTGTGAATGGCAAAACTGAAAATTTTGATGCAATCATCTTTGCAACTGGTTATAAGAGCAACGTGCCCTCATGGCTAAAG

>*AeYUC10*

ID: Unigene0098558

CDS:

CAACCACCACCGGTCATTATCGTCGGAGCTGGCCCCTCAGGCCTGGCCACCGCCGCTTCACTTAACCTCAATTCAATACCTTACATCATCCTCGAAAGAGAAGATTGTTCCGCTTCACTGTGGAAGAAATATTCATACGATCGTCTCCACCTTCACCTACACAAACAATTCTGCGAGCTTCCCCATTTCCCCTTCCCGGAATCCTACCCACGATTCATTTCAAAAGAACAATTCATAAGCTACTTGGACGATTACGTTTCTCGTTTTAAGATCACCCCTTTGTTCCGCCGATGCGTCGAGTCGGCCAAGTTCGATGAAGAGACCGAGGAATGGATCGTCAAGGCAAGGAACTTGGGTTCTAATGAGGTCGAGGAATTCAAAGGGAGGTTCTTAGCGGTGGCTAGTGGAGAAGCATGCGACCCTTATACGCCGGAGATCGAAGGATTGGAATCGTTCCCCGGTCTCGTTCTTCATTCAACTCAGTTCAGAAATGGGAAGTCATTTACGGATAAGAACGTTTTGGTTGTCGGGTCAGGTAATTCCGGCATGGAGATCGCGATGGACCTTGCGAATCACGGCGCCAAAACATCGATCGTTGTTCGAAGCCCGGTTCACATTCTTTCTAGGGAAATGGTGTTCTTGGGGTTGAATCTGTTGAAGTACATTCCATTGAACATGGTGGACTCCTTGATGATCATGCTTAGCAAGCTTGTTTACAGGGACTTGAGCAAGTACGGGATTAGCAGGCCTAAGGAGGGTCCATTTTTCATGAAAGTTGCGTATGGAAAGTACCCTGTTTTTGATGTAGGAACCTATAGTAAGATCAAGTCACAGGAGATTCAGGTGTTGCCTGCTATATCGAGTATACGAGGCAATGAGGTGGTGTTCGACGACGGCAGAGCACATGCTTTTGATACAATTGTGTTCTGCACTGGCTTCAAGCGGTCCACGCATATCTGGCTTAAGGAAAATCAAGATGGTTCCCTGCTGAACGATGACGGGATTCCTAAACCGAGTTTCCCGAACCATTGGAAGGGAAACAAGGGATTGTACTGTGTGGGTTTGTCTAGGAGAGGATTGTATGGTGCTAGCTTTGATGCACAGAACATAGCCAATGATATCAAATCCCTTCTC

>*AeTAR*

ID: Unigene0039279

CDS:

GGGGATCCGACAATGTACGAAAGCTATTGGCAAAAGATGGGGGAGGAGACCACCGTTGTTATTCCTGGATGGCAGTTCATGAGCTACTTTTCGGATGCCACCAGACTTTGCTGGTTCTTAGAGCCCGAGTTTGCGAACCAAATCGTCCGATTACATAATGTGGTTGGCAATGCAGTGACTGAGAACCGGCACATCGTTGTTGGAACCGGATCAACACAACTCTATCAGGCTGCCCTCTATGCGCTGTCCCCCGACACGGATGCAGAGCCCATCAGTGTGGTTTCAGCAGCCCCTTATTATTCTTCCTATCCTTTGATTACTGATTGCGTCAAGTCCAGGCTCTATAAGTGGGGAGGTGATGCCCGGAGCTTTAGTAAAAACGGGCCTTACATCGAACTCGTTACCTCCCCCAACAATCCCGATGGATTCACGAGGCGTTCGGTTGTGAACGGTAGTGAGGGAATACTAATTCATGATCTCGCCTATTATTGGCCACAATATACGCCCATATCTTCTGCAGCGAACTATGATTTGATGCTATTCACCGCCTCCAAGAGCACCGGTCATGCCGGGATGCGGATTGGGTGGGCTCTTGTTAAAGATGAAGATGTTGCAAGAAAAATGACAAAGTACATAGAGCTCAACACGATCGGTGTGTCGAAGGATTCACAGATGAGAGCAGCCAAGGTTTTGAAAGTTATATCCGACAACAGTGAAAGATCGAATGCAGGCGACTCTTTCTTCGAGTTCAGTTACCATGTTATGTCCAAGAGGTGGAAGCAATTGAGAGAGGCTGTGCATGAGAGTGGCCTATTTAGTGTGCCTGATTTTCCTCCCCAGCTCTGCAAGTTTCTCAACGTGGTCTTCGAGCCTCAACCTGCTTTTGCATGGGTAAAGTGTGAAGGAGAGATCGAAGACTGCGAAATCTTCCTCCGAGGGAAGAAGATATTGACTCGGAGTGGCAAACACTTTGGATTTAGCCCAAAGTACGTTCGAATAAGCATGTTGGATCGAGACCGGAATTATGATACCTTTGTAGAAAGATTGTCTACGATCCGATCA

>*AeSAUR71*

ID: Unigene0019619

CDS:

ATGAAGAATCTGATGCGCAGACTGTCTCGAGTTAAGGTGACTCACTCAACTCAATACTCAATGCTCCGATCTGAACCACCCGATCCTCGGTTGGGACCCGACAAGCAAGGATCGGACGTACCGCAGGGGCATTTCCCAGTTTACGTAGGGATTGGGATTGATCAGACGACGCCGACGCAGAGGTTCATCGTCAGTGCTGAGATGCTGCGGCAACCGATCTTCGTTGAGTTGCTGAACAGATCGGCCCAAGAGTACGGGTACGAACAAAGAGGAGTGTTGAGGATTCCGATTAACGCCGTCACCTTTGAACGCGTCGTTGAATCTCTCAGGCAAGGCCAACAACCTTTAAGCCTTGATGAACTT

>*AeDAO*

ID: Unigene0037376

CDS:

ATGGAGAAACCCCAGAGTATCCCAACGATTGATTTCTCAGATTTTCCGGCACAGTACGAGAAGCTAAGGAAAGCTTCGGAAGAGTGGGGTTGTTTCAGGGTGGTGAATCACAAGATTCCATTGGAATTGATGCAGGAAATGAAGAAAGTGGTGCGATCATTGCTTGACCTCCCCATAGAAATCAAGAGAAACAACACAGATGTCATCGCCGGCAGTGGCTACTGGGCTCCCAGTACCAAAAACCCACTTTATGAAGCTTTGGGTCTCTATGATATGGCATCCTCTCAAGCTGTTCATGACTTTTGTTCTCAGCTACAAGCCTCTCCTTCACAAAGAGATACAATTGAGAAGTATGCTGAAGCAATAAATAAGATGATCATGGATATAGGAGGGAAAATAGCTGAGAGTATGGGATTGAATGGAGATTACTGCAAGGAATGGCCTTGTCAATTTAGGATAAATAAGTACAATTTCACCCCAGAATCTGTAGGCACCACTGGGGTTCAAATACACACGGATTCTGGGTTTCTAACCATACTTCAAGACGATGAGAATGTTGGTGGTCTTGAAGTCGACAAATCTGGTGAATTTCTACCTGTTGATCCCTTGCCTGGCAGCCTCCTTGTCAATCTTGGAGATATGGCAAATGTGAGCTTAACACTCTTATGTGTCGATTCGATTTTTTTTCTTGTATGGTGAGCAGGTTGTTGTTGTTTTCTCGGTCAGGTATGGAGCAATGGAAGGTTACACACTGTGAGACATAGAGTGCAATGCAAGGAGGCAACGATTAGAGTGTCGATCGCCACATTCCTCTTGGGACCAAAAGAAGCAACAGTGGAGCCGCCGTCAGAACTGGTGGATTCCGACCACCCACGCCTTTACAAACCATTTACCTATGAGGAGTACAGAGAGCTCCGTATCTCCACTAAATTGCAAGCTGGTGAGGCTCTTGCACTCGTACGTTACAGC

>*AeMES*

ID: Unigene0019057

CDS:

ATGGGAAAGGAGGTGATGATGATGAGTATGGCAGAAAAGGAAACAGCAGCAGATAGGAATAACACACACTTTGTGTTGGTCCATGGTGTTGGTGGAGGAGCATGGTGCTGGTACAAAATCAGGTGTCTTATGGAGAATTGTGGGTACAAGGTTTCATGCATCGACCTCAAAGGCGCCGGGATTGATCGGTCTGATGCCGGTTCTATTCTATCTTTTGATGATTATAACCAGCCTCTCATGGACTTTATGTCTGCCTTGCCTGATACTGAAAAGGTGATATTGGTAGGTCATAGTGCTGGAGGGTTAAGTGTAACGCAAGCCACTCACAAATTCCCGGACAAAATCCGTCTGGCGGTTTATGTCGCAGCCACCATGCTAAAATCCGGTTTCGTGACTGATCAAGATGCCAAAGATGGAATACCAAATTTATCCCAATTCGGCGATGTGTACGAGCTAGGATTCGGATTGGGAGCGGAGCATCCTCCAACCAGTGCCGTTGTGAAAAAGGAATTCCAACGTAAAATCATCTATCAGATGAGCCCGCTAGAGGATTCAACCCTGGCAGCGATGCTTTTAAGGCCAGGACCGGTCCTAGCACTGTCGAGTGCTCGATTTAAAGAAGAGGGTGACTCTGTGGAAAAAGTTGGGCGGGTATACATTAAGACCATGCATGATAATGTTTTGAAACCGGAGCAGCAGGAGGCGATGTTGAAGAAATGGCCACCATCTCAAGTGTATGCTTTGGATAGTGACCACAGCCCCTTTTTCTCTGCTCCCTTTTCACTCTTTGGCTTACTCCTTAAAGTGGCAGCAACTTCGGCTGGATGCAAC

>*AeNCED*

ID: Unigene0006699

CDS:

TCTTCTTCATCAGCATCATCAGTTGCTAGTGGGAGCTGTTGTCTTAAGTATAAACTCCCTGTTTCATGCTCAAAAGCAGCTTCTTCTTCATCTGTAGGTTTCAAGAGGGGGAATGAGAGAAAATCCTACATTTCTTGTTCTCTTCAGACTCCTTCAATTCTTCATTTGCCTAAGCATCGGTCACCTGCTTTCCCTCCATCGTCTTCTTCTATTCCGACGACGAAACATGTTTCTTATGATGATGAGAAAGCTCAATCTCAGCAATGGAATCCATTGCAAAGAGCGGCGGCTATGGCTTTAGATGCAGTGGAGAATGCTTTAGTTTCTCACGAGCGTCGGCATCCTTTGCCGAAAACAGCTGACCCCAGAGTTCAAATTGCCGGCAACTTTGCTCCTGTTCCTGAGCAGCCGGTAAAGCAGAGACTCCCTGTTATCGGAACGATCCCGGAATGTATACAGGGTGTCTACGCTCGAAACGGGGCGAACCCGCTTCATGAGCCGGTGGCCGGACACCATTTCTTCGATGGGGATGGGATGGTTCATGCGGTTCAGTTCAAAAATGGCTCAGTTAGCTACGCTTGTAGGTTCACTGAAACGAACCGTCTTGTTCAAGAACGGTCTTTTGGGCGTCCGGTTTTCCCCAAGGCCATAGGTGAACTCCATGGCCACTCAGGCATAGCTAGGCTGTTACTTTTCTACGCTCGAGGCTTGTTCGGACTCGTTGATCCGAGCCATGGTACCGGTGTTGCAAACGCCGGGCTGGTTTACTTCAACGGCCACCTTCTCGCCATGTCCGAAGATGATTTGCCTTACCATGTTCGTATCACACCGTCCGGGGACTTGAAAACCGTCGGCAGATATGATTTTGATGGGCAGTTGAAGTCTGCAATGATTGCTCACCCCAAAGTTGATCCACAAACGGGTGAATTCTTTGCTCTTAGCTATGATGTTATTCAAAAACCATATCTTAAGTACTTCAAATTCGCACCCGATGGTACGAAATCACCCGATGTCGAAGTCCCTGTTGACGGTCCGACAATGATGCATGATTTCGCAATCACCGAGAACTTTGTGGTGATCCCGGACCAGCAAGTGGTGTTCAAATTGGGTGAAATGGTACATGGTGGTTCTCCCGTTGTGTATGACAAGGACAAGGTGTCTAGGTTCGGGGTGTTGGACCAGAATGCGGTTGATGCTACCGGAATTAAGTGGATTGAGGCACCTGATTGCTTTTGTTTCCATCTTTGGAATGCATGGGAAGAACCGGAGACCGATGAAGTTGTCGTGATTGGCTCTTGCATGACTCCCCCGGATTCGATTTTCAATGAATGCGAAGAGAATCTCAAGAGTGTCCTGTCCGAAATTCGATTGAATTTGAAGACTGGGAAGTCGACTCGCCGTGCCATCATTTCGGAATCCGAGCAAGTAAACTTGGAGGCAGGGATGGTGAACAAGAACTTACTAGGAAGGAAAACCCGCTATGCATATTTAGCTCTAGCTGAGCCTTGGCCTAAAGTGTCAGGATTCGCCAAGGTCGACCTTTCAACCGGCGAGGTTAAGAAGTATATCTATGGAGACAAAAGGTATGGCGGCGAGCCTTTGTTCTTTCCTCGAAACCCCGGTTCTCCGAATGAAGACGACGGCTATATCTTGGCTTTTCTTCACGATGAAAAGACCTGGAAATCAGAACTGCAGATAGTGAACGCCGTGGACTTAAGGCTAGAAGCGACGGTTCAACTACCGTCTCGAGTTCCATATGGTTTTCATGGAACATTCATAAGCTCAAAGGACTTGGAAAATCAGGCC

>*AePLY3*

ID: Unigene0087601

CDS:

ATGGTGACCGATGATTGTATCGACATGAACGAGAACAAGTTTAGTAAGATAGATGAAGAGTTCATCAAGAAATACCATAAACACGACGTTGAAGAAAATCAGTGCAGCTCTTCGCTCGTTAAACACATCAAAGCTCCGGTTCATCTCGTTTGGTCGTTGGTGAGAAGATTTGATCAACCACAGAAATATAAGCCATTTGTTAGCAGGTGTGTTCTACAGGGTGATCTTCAGATTGGAAGTGTAAGAGAAGTCAATGTTAAGTCGGGTTTGCCGGCCACAACTAGCAAAGAAAGGTTAGAGTATCTAGATGATGATGAGCATATTTTGAGCATGAAGATTGTTGGGGGAGACCATAGGTTGAAGAACTACTCATCCATTGTTACAGTGCATCCCGAGGTCATTGATGGCAGACCAGGAACATTGGTGATCGAGTCCTTTGTAGTGGATGTACCAGAAGGAAACACCGAGGATGAAACATGCTACTTTGTTGAGGCACTGATCAAGTGCAACCTCAAGTCATTGGCTGATGTTTCAGAGCGTTTGGCTCTTCAGGATCGGACTGAGCCAATTGAAAGAATG

>*AeZEP*

ID: Unigene0028677

CDS:

TTAGGTGGTAACAGCTCAAAACTTGAAGGAAGGTCTTTAAGTTGCAGACTGTCAGACAAGGCAAGTGACCAATTGAGGACATGGTTTGAAGATAATGATGCGTTGGAGCAGACTATTATTGGAGACTGGTACCTGTTGCCTGTTGGAGACGAAGTAGTTACTTCACAACCTACTTGTTTAAGTAGGGATGAGAAAAAAACTTTCATGATTGGGAGTGAAAAGAGCGATAACTTTTCGGAAACGTCAATAGTGATACCTTCGAAGCAGGTATCCAAAACGCATGCTCAAATCAGATATAAAGATGGTGCATTTTTCGTAGTTGATTTGCAGAGCGAGCTTGGAACCTTTATCGACGACGGAGGAAGAAGGTCGATGCTGTCCCCAAATGTTCCTACTCGTCTTCGTCCCTCTGATGTTATTGAGTTCGGTTCCGATAACAAGGTAGCACTCCGGGTAAAGGTGATGAGATCACATCCGAAGATGACTGAGAATCAAGCTGGTGGAATTCCTCAGGCA

>*AePLY9*

ID: Unigene0010234

CDS:

ATTAACGGCGGTGAAGCTTACGGTACGATGGAGGCGCAATACATACGGAGACATCACCGGCACGTCATCAGAGACAACCAGTGTTCCTCTGCTCTTGTCAAACATGTCAAAGCTCCTGTTAATCTCGTAACTCTCTGATTCATGTTTTTTTTCCTGAAAATTTTCAGAAAATAAATAAAAAATAAATAAATCTAAGAGTGTTTATTGTGGAAGGTATGGTCGTTGGTAAGGCGGTTTGATCAGCCGCAGAAGTATAAGCCGTTTGTGAGCAGGTGCATAATGAAAGGGGACCTCGGAATTGGTAGTGTTAGAGAAGTCAACGTGAAATCTGGTCTTCCGGCCACCACCAGCACAGAACGGTTAGAGCTTCTCGATGACGAAGAGCACATTCTCGGCATCAAAATCGTCGGCGGCGACCACCGTCTCAGG

>*AeAAO*

ID: Unigene0018208

CDS:

TGGGGCTCCATGGAAGTTGTTTTGTGTTGCATCCTTGCCTTGTCTTTGGTGCATGTTTCATTGGGTGCCAAAGCCAGGCATTTCAAATGGGAAGTGGAGTACATGTACAGATCTCCAGACTGTCTGGAACATATCGTGATGGGTATCAATGGTCAGTTTCCAGGACCAACCATTAGAGCTAAAGCTGGAGACACCATTGTTGTTGAACTCACGAACAAACTTCACACTGAAGGAGTTGTCATTCACTGGCATGGAATCAGACAGCTGGGAACACCATGGGCGGATGGGACTGCTTCCATTTCACAATGTGCTATCAACCCAGGAGAGACTTTCAAATACAGGTTCAAAGTGGATAGGCCCGGAACATATTTTTATCATGGACATTATGGAATGCAAAGATCTGCTGGACTGTATGGGTCCCTAATCGTGGACGTGGCAGATGGAGAAAAAGAACCCTTCCATTACGATGGTGAATTGAATCTATTGTTGAGTGATTGGTGGCACAAAGGTGTTCATGAACAAGAAGTTGGACTGTCATCCATTCCCTTTCGTTGGATTGGTGAACCACAGAGTCTTCTGATTAATGGCCGAGGGCAATACAATTGTTCCCTGGCAGCAAGATTTAGTAACCCACCGGTTGGCCAATGCAAGTTCAGAGGGAATGAACAATGCGCACCACAGATCCTGAAAGTCCAACCAAACAAAACCTACAGGCTCAGGATTGCCAGCACCACTGCTCTAGCTTCACTCAACTTGGCCATTGAGGGTCACAAAATGGTGGTAGTGGAAGCTGACGGGAACCATGTCCAACCGTTTGCTGTGAAGGATTTGGATATCTATTCTGGGGAAAGCTACTCAGTGTTGTTCAGAACAGACCAGAATCCTTCTAGGAACTACTGGATTTCAATCGGTGTTAGAGGACGAGAACCCAAAACAAGTCAAGCCCTCACCATTTTGAACTATTCCCCCACCTCTGCATCAAAGATCCCCACGTCTAGGCCCCCGGTAACACCTCCATGGAACGATTACAACCACAGCAAGGCATTCACTAAAAGCATATACGCCCTCATGGGGTCACCCAAGCCACCCAAAACGTCTCACCGTCGGATCGTGCTACTCAACACTCAGAACCGGGTCAATGGATTCATCAAGTGGTCGATCAACAATGTATCTTTGGTGTTACCATCCACTCCTTACTTGGGGTCCATTAAATTTGGTCTAAACCATGCTTTTGACCAAAGAAGCCCACCTGACAACTATGACAGCAGCTATGACATCATGAAGCCTGCAGTGAACCAGAATTCAACTCAAGGGAGTGGGATTTACACCATCACCTTAAACACCACGGTTGATGTGATTCTCCAAAACGCCAATGCACTGGCAAAGGATCTGAGTGAGATACATCCGTGGCATTTGCATGGCCATGATTTCTGGGTGTTGGGATATGGGGAAGGGAAGTTCAAAGATGGAGACGAGAAGACATTTAACTTGAACAACCCACCATTAAGGAACAGTGCTGTGATTTTCCCATATGGATGGACTGCATTGAGGTTTGTTGCTGATAATCCAGGGGTGTGGGCATTCCATTGTCACATCGAACCACATTTGCATATGGGGATGGGGGTTGTTTTTGCTGAAGGAGTCGATCGTGTTGGTCAAATACCAAGAGAAGCTATTGCTTGTGGATTGACAGGAAACAAGCAGAAC

>*AeABF*

ID: Unigene0062393

CDS:

TCGTTGACATTGCCGAGGACTCTGAGTCAGAAAACAGTTGAAGAAGTATGGAAAGACTTATTTAAGGAGAATGATGGTGCTAAAAATGTAAGTAATGGCAGTGGTGCTGTTGTTGGTGGAGCAAATTTGCCGCAAAGGCAACAGACATTGGGAGAGGTAACTTTGGAGGAGTTCTTGGCGAGGGCAGGTGTAGTAAGAGAAGATACCCAAAAGATTGGAATGTCAAATAACACTGGATTCTTTGATAACAACTCTGGTTTAGCACTTCGGTTTCAACAGAATATTGGAAGTAATGCTTTTTTGAGTAACAATAGCTCAGCTCTTAATCACTCTCCAAGGCTACCATTAAACACGAATGGAGCCAAATCATCACAACCACAGCATCAGCATCAGCAGCAACAACAACAGCCGATCTTCCCTAAACAACCAACAGTTGCATGTGGTCCATCTATGCACTTGATACACACTACACAATTTGCTACTCCTGGAGCTCGGGGTTCAGTAGCATTGAATATTAATCTAGTTCAATCTACCGGGCTGCAGAGTCGTGGGATGGGAATAGTTGGTTTAGCATCTCCTGCAAACCATATATCTTCAGATGTGATTTCAAGCAACAGCATAGATACTTCGTCTTTATCACCAGTTCCTTATGTGCTTGGTCGGGGAAGAAAACGCAGTGCAGCTTTGGAGAAAGTAGTTGAGAGAAGGCAAAGGAGAATGATTAAGAAC

>*AeCYP707A*

ID: Unigene0001233

CDS:

ATGGCTTTCCAGTTCCTGCTCCCCTTGCTTGCTTCCCTCCTCTTCCTTTTCCTCTTCCGTTGTTTACTTAAATTCTTCAATTCCAGCAACCGCCAATTGCCCCTCCCGCCGGGAACCATGGGTTGGCCATACATCGGCGAAACCTTCCAGCTTTACTCCCAGAACCCAAATGTCTTCTTTGCTTCAAAGCAAAAGAGGTACGGTTCCGTATTCAAGACCCATATACTCGGGTGTCCCTGCGTGATGATTTCCAGCCCAGAAGCGGCTAAGTTCGTGCTCGTCACCAAATCTCATCTGTTCAAGCCGACGTTTCCTGCCAGCAAAGAGAGGATGTTAGGCAAACAAGCCATTTTCTTCAGTCAAGGACAGTACCATGCTCAATTGAGGAAGCTTGTACTTCGTTCTTTCATGCCCGAAGCCATCAAAACCATCATCTCTAACATTGAATCCATTTCCAAAGACTCCCTCAACTCTTTGGAAGGGAGGCTGATTACCACTTTCCAAGAAATGAAAACATACACGTTCAATGTCGCTTTGCTATCGATATTCGGAGAGGATGAAGTTCTGTACAGAGAAGATCTCAAGAGATGCTACTACGTTCTAGAGAAAGGGTATAACTCGATGCCCATTAATGTCCCTGGTACACTCTTCAACAAGTCGATGAAAGCTAGGAAAGAGCTTGCTCAGATCCTGGCCAAAATCATATCAACCAGGAGGCAAATGAAGCAGGACTGCAATGACTTGTTGGGGTCTTTCATGGGTGATAAAGAAGGCCTCACTGATGAACAAATCGCCGACAACATTATCGGCGTAATCTTTGCCGCTCGTGACACTACCGCCAGCGTACTCACATGGATAATTATGTACCTAGGTGAAAACCCCAGCGTTCTTAAAGCTGTCACGGATGAACAAGAGGCTATAATACGAGAGAAAGAGAAATGCGGCGAGGAACAAAGCCTGAGCTGGGCTGATACTAAGATGATGCCAATTACTTCAAGGGTGATTCAGGAGACACTTAGGGTTGCTTCGATTTTATCGTTTACCTTCAGGGAAGCAGTGGCCGATGTTGAATATGAAGGTGAGTTACACCAGCTGCTGCTAGTAGCCTAGTACTACTACCTTGCCCTTTCCCATGCCTAATTGTTAGTGTAGTTTTATCCTATGCCTTTGGTGCTAATAGCCTGACTTGATTTCTGTAAAAAACCAGGTTATCTTATACCAAAAGGATGGAAAGTTTTACCACTTTTTAGAAACATTCATCACAGCCCAGAAATCTTCCCAGATCCTGAAAAGTTTGATCCTTCAAGATTTGAGGTGGTTCCCAAACCCAATACATTTATGCCATTTGGCAATGGAACCCATTCATGTCCCGGGAATGAGTTAGCCAAGCTGGAAACCCTGATCTTTCTCCATCATCTGACCACAAAGTACAGGTAAATTTAATTTGTTGACAGCCTTGAAATCCTTTTCTGTTTTATGCTTTGCCTGTTTCTATTAATAGGAAGAGAAAAAACATATCTTTTCTGATATGAATATATTATGGGATTTTGCAGGTGGTCTGTGGTAGGTACAAATAGTGGGATTCAATATGGTCCTTTTGCTCTCCCCCAAAATGGTCTGCCCCTCAGATTAATCAGAAAATCA

>*AeALDH1*

ID: Unigene0015352

CDS:

ATGGCAACTCGCAGAATCTCTTCACTGCTTACTCGTTCACTCTCTGCTTCTTCCTCTGTCTCTACTTCTCTGCTTTCATCTCTAGGGAAAAGCAGCGGCAGGAATGCATGTGTGAGGTTTGGCAATGCTGCGCTTCTGGAAGATCTGGTTGTTCCACCGGTTCAAATATCCTACACTCAGCATTTAATTGATGGGAAATTTGTAGATGCTGCATCCGGGAAAACATTCCCTACTTATGACCCTCGTACCGGAGAAGTGATTGCACATGTTGCTGAAGGGGATGCCGAAGATATTAACCGAGCAGTGGCCGCTGCTCGCAAGGCATTCGATGAGGGACCATGGCCGAAAATGAGTCCCTATGAACGTTCACGGATACTGTTGCGGTTTGCCGATTTGGTCGAGAAACATAGTGAGGAGCTCGCTGCCTTGGAGACATGGAATAATGGAAAGCCTCATGAGCAGGCCGCTAAATCTGAATTGCCAATGCTTGTTCGCCTTTTTCACTACTATTCCGGTTGGGCAGATAAGATCCATGGTCTCACAGTTCCGGCTGATGGACCTCACCATGTTCAGACATTGCATGAGCCTATAGGTGTTGCAGGACAGATAATTCCATGGAACTTTCCCCTTCTCATGTTTGCTTGGAAGGTTGGGCCTGCATTGGCATGTGGCAATACCATAGTCTTAAAGACTGCTGAGCAAACTCCTTTGACTGCTCTCTATGTGGCTAAGCTGTTCCATGAGGCTGGTCTCCCTCCAGGTGTTCTGAATGTAGTTTCTGGCTATGGTCCAACAGCTGGTGCTCCTCTTGCCAGCCACATGGATGTAGACAAGGTTGCATTCACCGGATCAACCGATACTGGTAAGGTTGTCCTTGAGTTGGCTGCAAGAAGCAATCTTAAACCAGTGACATTAGAGCTTGGAGGGAAATCACCTTTCATAGTATGCGAAGATGCTGATGTTGATAAAGCTGTGGAGCTTGCTCATTTTGCTTTATTCTTTAATCAG

>*AeALDH2*

ID: Unigene0009490

CDS:

ATGGCGGTTCAAATCCCGAGTCGACACTTATTCATCGATGGCGAGTGGAGAGAGCCGGTTCTCAAGAAGAGAATCCCAATCATCAACCCTGCTACTGAACAGATCATCGGTGATATCCCGGCAGCTACATCTGAAGATGTGGACCTTGCGGTTGCCGCTGCTCGGAGAGCCCTTTCTAGAAACAAAGGAAAGGATTGGGCTTCAGCTTCTGGCGCTGTTCGTGCCAAGTATTTACGTGCCATTGCTGCTAAGATAACAGAGAGAAAAACTGAATTGGGTAAGCTTGAAGCAATGGATTGTGGGAAACCGCTTGATGAAGCAGTTTGGGACATGGAGGATGTTGCTGGATGTTTCGAGTATTATGCAGACCTTGCTGAAGGGTTAGATGCAAAGCAAAAGGCTCCTGTCTCT

>*AePAO1*

ID: Unigene0006927

CDS:

GGTATTTGCTATCCAAATGGGGTGAGGAGGCAAGCAAGATCACCGTCAGTGATCGTGATTGGGGCTGGAATTGCCGGAATAACTGCTGCTCATGCTCTCCGTGAAGCCTCAATTCAGGTTACGGTGTTAGAATCCAGGGACAGAATTGGTGGTCGAGTTCATACAGATTACTCATTTGGTTTTCCTGTTGACCTTGGTGCTTCTTGGTTGCACGGAGTTTCCAAAGAAAATCCATTGGCACCATTGATCAGTAGACTTGGACTCCCACTTTATCAGACTAGTGGTGACAACTCTGTGCTGTATGAACATGACTTGGAGAGCTACGCACTCTTTGATATGGATGGTCGTCAAGTTCCACAGGAGTTGGTCACTAAGGTTGGAGAAGCATTTGAATGCATTTTACAAGAGACAGATAAAGTAAGAAAAGAGCAGAGTGAAGATATGTCCATAACTCGTGCTTTCTCAATCGTTTTTGAAAGAAGACCAGAATTAAGGTTGGAAGGGCTTGAACATAAGGTACTTCAGTGGTACATATGTCGTATGGAAGGTTGGTTTGCTTCAGATGCCGATACTATCTCACTTAAAAGCTGGGACCAGGAAGAGCTGTTACCTGGTGGTCACGGACTCATGGTCAGGGGATATCTTCCTGTCATAAACACACTGGCCAAAGGTATTGACATCCGCTTGAACCACAGGGTTACAAAAATAGTGAGGCGTTACAATGGAGTGAAGATTACTGTGGAAGATGGAACTACATTTGTTGCAGATGCTGTTATTGTTGCTGTTCCTCTAGGTGTACTAAAAGCCAAGACCATCAAGTTCGAACCAAAGCTTCCTGAATGGAAGGAAGCAGCAATTGATGATCTTGGAGTGGGAATTGAGAACAAAATAATATTGCACTTCGACAAGGTGTTTTGGCCTAATGTGGAGTTTTTGGGAGTTGTAGCTGAGACATCATACGGTTGCAGCTACTTTCTAAACCTTCACAAGGCCACAGGTCACCCCGTCCTTGTGTATATGCCTGCAGGACAGCTGGCCAGAGATATTGAGAAATTGTCTGATAAAGCTGCAGCAGAGTTTGCTTTTATGCAACTGAAGAAGATCCTTCCAGAGGCATCTGCCCCGATTCAGCATCTTGTTTCTCGGTGGGGATCAGACGTTAATTCACTTGGTTCCTATAGCTATGATGCAGTAGGCAAGCCCCATGATCTGTATGAGAAGCTAAGGGTCCCAGTGGATAATGTCTTCTTTGCTGGGGAGGCAACCAGTATTAGCTACCCAGGGTCCATTCATGGTGCATTTTCAACGGCTCAGATGGCTGCCGAGGCTTGTAGGATGCGTGTACTGGAACGATATGGAGAGTTTGACTTGTTCCAGCCAGTCATGGGGGAGGAAGGAGGATTTTTTGTCCCGCTCTTGATCACCCGTTTG

>*AePAO2*

ID: Unigene0035208

CDS:

ATGGCCAAGAAGCCGAGAATTGTGGTAATTGGTGCAGGCATGGCTGGTCTTACAGCGGCCAACAAGCTTTACACTTCAACTGGTTCAAAAGACATGTTCGAGCTGTTTGTTGTTGAAGGTGGTAATAGAATTGGAGGAAGAATCAACACTTCCGAGTTTTGTGGTGATCGGATCGAGATGGGAGCTACTTGGATCCATGGCATAGGAGGCAGCCCGGTTCATCAAATTGCTGAAGAAATCAATGCATTGGAGTCTGATCAGCCATGGGAGCGTATGGATGGATTCCCTGATGAGCCTAAGACCATTGCCGAAGGCGGTTTCGAGCTGAATGCTTCCGTTTTCGGTCCGGTATCGTGTCTTTTCAAGAACCTGATGGACTTTGTTCAGAGGAAGGAGACTGAAGACAGTGTTGTAGGCGGCTTCGGCAATGAGAGCGTCGGTGCTTATCTTCGACAAGGCCTTGATGCGTATTGGGATGCTCGGAAGGAAATTGAAGAGCTTAAAGGATATGGAGAATGGAGCAGGAAGTTGCTTGAGGAAGCTGTTTTCGAAATGCTGGAGAACACACAGAGGACTTATACTTCGGCCGGTGATCTTTTCGATCTAGATTCCGAAGCAGAAAGTGAGTACCAGATGTTCCCTGGTGAAGAAATCACCATTGCTAAAGGCTACTCGAGCATAATCAAACATCTTGCATCGGTTCTACCGTCCGGTGTCATCCAATTAGGCCGCAGAGTCGACAGAATTGAATGGCAACCACAGGGTAATCATCATCATAATCAACAATTAGACGATTCGAGGCCGGTGAAGATACATTTTGTCGATGGATCGTATATGTTGGCCGATCATATTATAGTCACCGTTTCATTAGGGGTCTTGAAAGCTGGAACTTGTAAGGATTCAGGTATGCTCTTCAGTCCTCCCTTGCCTTCTTACAAGACAGATGCTATATCAAGACTTGGATATGGTGTTGTTAACAAGTTGTTTCTCCAATTAAGTCCGGATGGTAATAAAAAAACAGAGTTCCCTTCCCTGCAAATGGTGTTCCATCGTTCGGAATCCGAGTTACGTGACAAAAAGATCCCATGGTGGATAAGGAGGACAGATTCTTTGTCCCCTATTTACAACAATTCAAGTGTGCTCCTATCTTGGTTTGCAGGGAAAGAAGCTCTCCATCTCGAAACACTTAGCGACAAAGAGATTATAGACACGGTATCGACAACGGTTTCGAGTTTACTATCGAAGCCCCATAGAGAAATGAAGTCTGAGAATGGCTTCAAATCCAATGGATTCTGCAATGGTAATGAAAGTGAAAGTGAAAGTGAAGTGAGATTCATTGAGGTTTTGAAGACCAAATGGGGAAGTGATCCACTGTTCTTGGGGTCTTACAGTTACGTCGCGGTTGGATCGAGTGGTGCCGATTTTGACACAATGGCCGAGCCATTACCAAAGTCACCTGCCGGTACCATGCATCATCATCATCCACTTCAAATTTTGTTTGCTGGGGAAGCTACACATAGAACTCACTATTCCACAACCCATGGAGCTTACTTCAGTGGTCTAAGAGAAGCCAATAGGCTTCTCCAACACTATCATTGTGTTGAA

>*AeGAD1*

ID: Unigene0045486

CDS:

ATGGTGATCACAACCGCGAATCCAGAAGCAGGGCAGTATGTTCAATCCAGTACTTTTGCTTCAAGATATGTCCGCGAGCCACTTCCTAGATTCAAGATGCCAGAAAATTCAATGCCCAAGGAAGCAGCCTATCAAGTGATAAACGATGAGCTGATGCTTGACGGAAATCCGAGGTTGAACCTGGCATCATTTGTGACCACATGGATGGAGCCCGAGTGTGATCAACTCATGATGGCTGCCATAAACAAGAACTATGTTGACATGGATGAATACCCTGTTACCACTGAGCTCCAGAATCGATGTGTAAACATGATAGCCAACTTATACCATGCTCCAATTGGAAACAATGAGACAGCGGTCGGTGTCGGGACCGTTGGTTCCTCCGAGGCAATAATGCTTGCAGGCTTAGCCTTTAAAAGAAAGTGGCAGCAAAAGAGAAAATCACAGGGGAAACCATATGATAAGCCCAACATAGTCACAGGAGCGAATGTGCAGGTTTGCTGGGAGAAGTTTGCAAGGTACTTCGAGGTCGAGTTGAAGGAGGTGAAGCTGAGAGAGGGATATTATGTGATGGACCCTGTGAAAGCAGTTGAAATGGTCGACGAGAACACCATATGCGTCACAGCCATTCTTGGATCTACCCTGACCGGGGAGTTCGAGAATGTGAAGCTCCTTAATGAACTTCTTACAAACAAAAACAAGGAAACTGGTTGGGATACACCTATACATGTGGATGCTGCCAGTGGAGGGTTCATTGCTCCCTTTGTTTACCCTGATCTCGAATGGGATTTCCGCCTGCCATTAGTAAAAAGCATTAATGTCAGCGGGCACAAGTATGGTCTTGTCTATGCCGGTGTCGGTTGGGTCGTGTGGAGAAGCAAGGACGATTTGCCCGACGATCTTGTCTTTCACATCAACTACCTTGGATCCGATCAGCCTACTTTCACCTTAAACTTCTCGAAAGGCTCTAGTCAAATCATAGCTCAATATTATCAGCTTATCCGGCTCGGTTTCGAG

>*AeGAD2*

ID: Unigene0076507

CDS:

GCCTACACCATGCCTGCCGACGCTCAGCACATCACTGTACTCCGTGTGGTCATCAGGGAAGATTTCTCCCGCACCTTGGCCGAGCGTCTCGTCAACGACATTGAGAAAGTGCTGCACGAGCTTGACACCCTCCCCGCGAAGGTCACGGCCAAACTGGCTATGGCTGAAGAAGCAGAAACCCATAACGGCACTGTTAAGAAGACCGATATTGAGACTCAAAGTGAAGTCATGACTTACTGGAAGAAGTATGTGAGTGAGAGAATATCCAGCAAGAACAAAATTTGT

>*AeACT*

ID: Unigene0004305

CDS:

CGTCCTCGTCACACTGGTGTTATGGTTGGGATGGGTCAGAAGGATGCCTATGTAGGAGATGAGGCACAATCTAAACGAGGTATCTTGACATTGAAATATCCTATTGAGCATGGTATTGTTAGCAACTGGGATGATATGGAAAAGATCTGGCATCATACTTTCTACAATGAGCTTCGTGTTGCTCCCGAGGAGCACCCTGTGCTTCTCACTGAGGCACCTCTTAACCCCAAGGCCAATAGAGAAAAGATGACCCAGATCATGTTTGAGACCTTTAATGTACCTGCTATGTATGTTGCCATCCAGGCTGTTCTCTCTTTGTATGCTAGTGGTCGTACAACAGGTATTGTGCTGGATTCTGGTGATGGTGTGTCTCACACTGTACCCATCTATGAAGGTTATGCCCTTCCGCATGCCATCCTCCGTCTAGACCTTGCTGGTCGTGATCTCACTGATTCTTTGATGAAGATTCTTACTGAGAGAGGTTACATGTTCACCACCACTGCTGAACGGGAAATTGTCCGTGACATGAAGGAAAAGCTAGCTTATGTTGCCCTTGACTATGAGCAAGAGCTG
